# A Comprehensive Analysis of Runs of Homozygosity of Eleven Cattle Breeds Representing Different Production Types

**DOI:** 10.3390/ani9121024

**Published:** 2019-11-25

**Authors:** Tomasz Szmatoła, Artur Gurgul, Igor Jasielczuk, Tomasz Ząbek, Katarzyna Ropka-Molik, Zygmunt Litwińczuk, Monika Bugno-Poniewierska

**Affiliations:** 1 University Centre of Veterinary Medicine, University of Agriculture in Kraków, Al. Mickiewicza 24/28, 30-059 Kraków, Poland; artur.gurgul@urk.edu.pl (A.G.); igor.jasielczuk@urk.edu.pl (I.J.); 2 Department of Animal Molecular Biology, National Research Institute of Animal Production, Krakowska 1, 32-083 Balice, Poland; t.zabek@izoo.krakow.pl (T.Z.); katarzyna.ropka@izoo.krakow.pl (K.R.-M.); 3 Sub-Department of Cattle Breeding and Genetic Resources Conservation, University of Life Sciences in Lublin, Akademicka 13, 20-950 Lublin, Poland; zygmunt.litwinczuk@up.lublin.pl; 4 Department of Animal Reproduction, Anatomy and Genomics, University of Agriculture in Kraków, al. Mickiewicza 24/28, 30-059 Kraków, Poland; Monika.Bugno-Poniewierska@urk.edu.pl

**Keywords:** runs of homozygosity, autozygosity, microarray, cattle

## Abstract

**Simple Summary:**

Runs of homozygosity (ROH) regions are known to be common in the genomes of cattle and have become a subject of interest of various research in recent years. ROH can be used as a valuable tool to estimate inbreeding, which needs to be controlled in livestock populations. Moreover, analysis of ROH is considered to be an effective method of identifying genome regions that are a subject of selection pressure, which may help in understanding the genetic aspects of production traits under selection. In this study, we analyzed ROH characteristics of 11 cattle breeds, both commercial and native, maintained in Poland. We presented distinct differences in the length, quantity and frequency of ROH between the analyzed breeds as well as in the levels of genomic inbreeding. Higher levels of inbreeding were characteristic for commercial breeds, especially beef breeds. In addition, within ROH islands, we observed a number of genes with a confirmed influence on the level of production traits. The presented results and identified genes can be a basis for further research focused on the identification of genes and markers essential in the determination of the most important production traits in cattle.

**Abstract:**

In the presented research, BovineSNP50 microarrays (Illumina) were applied to determine runs of homozygosity in the genomes of 11 cattle breeds maintained in Poland. These cattle breeds represent three basic utility types: milk, meat and dual purpose. Analysis of runs of homozygosity allowed the evaluation of the level of autozygosity within each breed in order to calculate the genomic inbreeding coefficient (F_ROH_), as well as to identify regions of the genome with a high frequency of ROH occurrence, which may reflect traces of directional selectin left in their genomes. Visible differences in the length and distribution of runs of homozygosity in the genomes of the analyzed cattle breeds have been observed. The highest mean number and mean sums of lengths of runs of homozygosity were characteristic for Hereford cattle and intermediate for the Holstein-Friesian Black-and-White variety, Holstein-Friesian Red-and-White variety, Simmental, Limousin, Montbeliarde and Charolais breeds. However, lower values were observed for cattle of conserved breeds. Moreover, the selected livestock differed in the level of inbreeding estimated using the F_ROH_ coefficient. In regions of the genome with a high frequency of ROH occurrence, which may reflect the impact of directional selection, a number of genes were observed that can be potentially related to the production traits which are under selection pressure for specific production types. The most important detected genes were *GHR*, *MSTN*, *DGAT1*, *FABP4*, and *TRH*, with a known influence on the milk and meat traits of the studied cattle breeds.

## 1. Introduction

Runs of homozygosity (ROH) can be defined as long homozygous chromosomal regions within which both haplotypes inherited from parents are identical. The identity of large chromosome segments inherited from both parents by the offspring may be the result of their descent from a common ancestor and point to a certain level of parental relatedness [1]. Over the past 20 years, there has been a rapid development of high-throughput genomic analysis methods, including next-generation sequencing (NGS) and genotype-based microarrays (SNPs). Both of these methods allow for effective identification of runs of homozygosity [2]. However, SNP microarrays remain the most popular tool for determining ROH, mainly due to their lower costs and less time-consuming analysis compared to NGS.

ROH can be used as a valuable tool to estimate inbreeding, which is one of the methods often used in animal husbandry utilized to preserve characteristics of outstanding individuals in the population [3]. The inbreeding coefficient determined using ROH (called F_ROH_) is calculated as the ratio of the total length of ROH for each individual in the selected ROH length category to the total length of the autosomal chromosomes covered by SNPs [4]. The determination of the ROH-based inbreeding coefficient shows several advantages compared to the classical inbreeding coefficient calculated on the basis of pedigree data (F_PED_). F_ROH_ more accurately predicts the actual level of autozygosity of the genome and it can be estimated in any animal with genotypic data—even if information about its pedigree is not available [1,3,5].

An increase in the homozygosity of certain regions of the genome may also take place as a result of intense artificial selection, which leads to the increase of frequency of beneficial alleles in the population in a process similar to genetic drift [6,7,8]. Identification of such regions that show a reduction or elimination of polymorphisms (known as selective sweeps) may indicate the occurrence of directional selection and help in understanding the genetic aspects of selected production traits [9,10]. Analysis of the frequency of alleles and genomic homozygosity are considered to be effective methods of identifying genome regions that are a subject of selection pressure [10,11]. Therefore, it is assumed that the size and location of ROH may be correlated with the effect of selection pressure and that ROH should occur non-randomly in the genome [12]. Moreover, it has been shown that ROH can create unique patterns in the genomes of different breeds, probably under the influence of natural or artificial selection [13,14]. This information can be potentially used in evolutionary research as well as in gene mapping related to production traits [15]. In the research of Nothnagel et al. [13] and Pemberton et al. [14], it has been shown that ROH are not evenly distributed throughout the genome and form so-called “ROH islands”. These regions are widely observed in populations and form specific patterns within the genome. These patterns can be used as a useful tool to identify the phenomenon called “selective sweeps” and genome regions subject to selection pressure [10,11,12,16].

The purpose of this study was to characterize runs of homozygosity and identify ROH patterns in the genomes of 11 selected cattle breeds (Holstein-Friesian Black-and-White variety (HO), Holstein-Friesian Red-and-White variety (RW), Simmental (SM), Limusin (LM), Hereford (HH), Charolais (CH), Montbeliarde (MO), White-Backed (BG), Polish Red (RP), Polish Red-and-White (ZR) and Polish black and white (ZB)) maintained in Poland. As part of the study, genome regions were identified that are characterized by a high incidence of ROH, i.e., potentially under the influence of directional selection. Moreover, the structure of homozygosity sequences and their patterns was determined in the examined cattle breeds, which will contribute to a better understanding of genetic differences between the breeds, their breeding history and changes in the genome under the influence of intensive selection. In addition, the aim of this study was to assess the inbred of the studied animal populations based on the inbreeding coefficient calculated based on homozygosity sequences (F_ROH_). This is particularly important in the case of animals belonging to herds of conserved breeds, for which pedigree information is often shallow and the level of inbreeding is crucial for ongoing programs for the protection of genetic resources.

## 2. Materials and Methods 

### 2.1. Research Material, Genotyping and Data Filtering

The research material was genomic DNA obtained from 1931 randomly selected cows and bulls belonging to 11 breeds of cattle kept in Poland. For meat and milk production type breeds, the material was randomly sampled from Biological Material Bank of National Institute of Animal Production which included samples shipped by breeders for parentage verification. For conserved breeds, animals were randomly sampled from small individual farms located in various regions of Poland among the animals included in conservation program. In both cases, animals were controlled not to include closely related individuals. The detailed characteristics of cattle regarding the use type and the number of individuals of a given breed are presented in Table 1 and additional information regarding the conserved breeds is presented in Supplementary Material S1.

Some of the animals from four of the selected breeds (HO, RP, LM and SM) were previously analyzed in our earlier publication [17]. However, the results described in our previous publication were maintained on a different SNP dataset (different filtration of microarrays) and the ROH identification was maintained in a different way. Moreover, all the animal procedures were approved by the Local Animal Care Ethics Committee No. II in Kraków—permission number 1293/2016 in accordance with EU regulations. 

DNA was isolated from semen, ear tissue or whole blood using Sherlock AX (A & A Biotechnology, Gdynia, Poland) or QuickGene DNA whole blood kit S (KURABO, Kurashiki, Japan) kits. The purity and concentration of the obtained DNA was determined using a NanoDrop2000 spectrophotometer. High-quality DNA was normalized to the required concentration (50 ng/μL) and analyzed using Illumina BovineSNP50 v2 BeadChip microarrays (Illumina Inc., San Diego, CA, USA). Finally, the microarrays were scanned using the HiScanSQ system (Illumina). All the procedures were carried out in accordance with the manufacturer’s protocol. 

Filtering of the genotypic data obtained after scanning was performed together for all the tested breeds while ROH identification was performed separately for each examined breed. The dataset contained 54,609 SNPs before filtering. Only animals with more than 95% genotypes (CallRate) and SNP with GenCall quality coefficients above 0.7 and GenTrain above 0.4 were used for further analysis. Next, SNPs on the X, Y chromosome and those without a fixed genomic position (mapped to contigs) were removed. The final marker panel included probes for 42717 SNP markers localized in the genome with a mean distance of 55.4 kb (±44.4 kb).

### 2.2. Identification of ROH

The SNP panel obtained after filtering was used to identify ROH for each animal individually using cgaTOH software [18]. The following parameters were used for the identification of ROH: a minimum number of 30 consecutive homozygous SNPs in ROH with a maximum distance between SNPs equal to 1 Mb. The identified ROHs were assigned to five length categories: 1–2 Mb, 2–4 Mb, 4–8 Mb, 8–16 Mb and above 16 Mb. To calculate the number of heterozygotes to be included in the identification for each ROH length category, ROH was initially determined without allowing any heterozygotes. This enabled the calculation of the number of SNPs for each category of ROH length. Based on this data, assuming a 0.2% genotyping error for the Illumina microarrays [19], 0 heterozygotes were assigned in the first four ROH length categories (1–2 Mb, 2–4 Mb, 4–8 Mb, 8–16 Mb) and 1 heterozygote for the last ROH category with a size above 16 Mb. The number of missing genotypes that were allowed in ROH was calculated according to the methodology proposed by Ferenčaković et al. [20] and the following criteria were applied: in the categories of ROH 1–2 Mb and 2–4 Mb, no missing SNP genotypes were allowed, in the category 4–8 Mb, one missing SNP, in the category 8–16 Mb, two missing SNPs, while in the category over 16 Mb, four missing SNPs were allowed. The average sums of ROH in selected categories were calculated by summing all ROHs identified for each animal in each category and averaging the results within the breed.

### 2.3. Determination of Inbreeding Coefficient Based on ROH

The F_ROH_ inbreeding coefficient was calculated according to the methodology proposed by McQuillan et al. [4] by dividing the total ROH length for each individual in the selected ROH length category by the total length of autosomal chromosomes covered by SNPs (2510.6 Mb). The ROH length categories were as follows: >1 Mb, >2 Mb, >4 Mb, >8 Mb and >16 Mb.

The literature data show that F_ROH_ calculated for ROH above 1 Mb is the most frequently used measure of genomic inbreeding and best describes recent animal relatedness. However, due to some errors in the identification of short ROH observed in the case of 54 K microarrays [1], F_ROH_ for ROH with length above 4 Mb was also calculated to compare the obtained coefficients and eliminate errors resulting from the use of short ROH segments for calculations.

### 2.4. Identification of Genome Regions with a High Frequency of ROH Occurrence 

To identify genome regions characterized by a high frequency of ROH occurrence, result files determining how many times each SNP appeared in a given population in ROH were generated for each population. Subsequently, 1% of the highest occurrence values were chosen as the threshold value above which the identified genome regions were classified as “ROH islands” in a given population. These regions were analyzed for overlapped genes using the UCSC Genome Browser tool [21] based on the UMD3.1 bovine genome assembly and later, using the Panther Classification System [22] to identify their molecular functions and related biological processes. In addition, the size, range and frequency of the occurrence of ROH between the studied breeds was compared and the genomic ROH patterns were checked for similarities between the individual breeds and separate production types.

## 3. Results

### 3.1. Characteristics of Runs of Homozygosity

One of the most important parameters characterizing the structure of ROH is the number and average sum of lengths of ROH per animal. The highest mean number of ROH per animal in individual populations were observed for the Hereford breed (80.6), while the correspondingly lower mean values were characteristic for conserved breeds: White-Backed (23.9), Polish Red (23.3) and Polish Red-and-White (21.8). The Holstein-Friesian Black-and-White variety, Holstein-Friesian Red-and-White variety, Simmental, Limousin, Montbeliarde and Charolais were characterized by a slightly higher number of ROH relative to conserved breeds with an average number of ROH in the range of 38.8 to 53.3 per animal.

The same trend was observed in the case of average sums of ROH lengths for individual animals. The highest average ROH lengths were observed for Hereford breed (378.0 Mb) and the lowest for conserved breeds—White-Backed (127.6 Mb), Polish Red (131.7 Mb), Polish Red-and-White (105.7 Mb) and Polish Black-and-White (135.8 Mb). HO, RW and MO breeds were characterized by mean values of sums of ROH lengths in the range from 216.7 to 295.1 Mb, while SM, LM, and CH in the range from 147.4 to 163.2 Mb.

**Figure 1:**
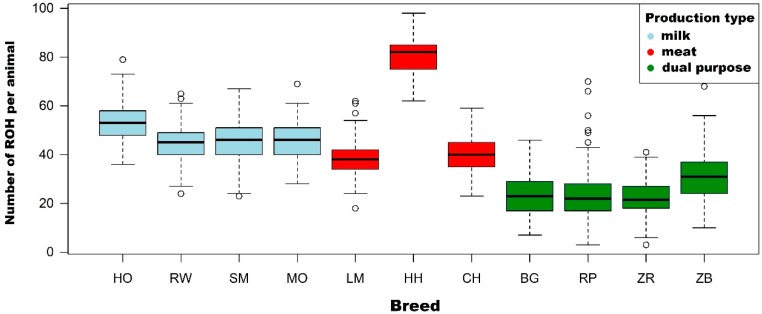
The number of runs of homozygosity (ROH) per animal with division into breeds and cattle production types.

**Figure 2:**
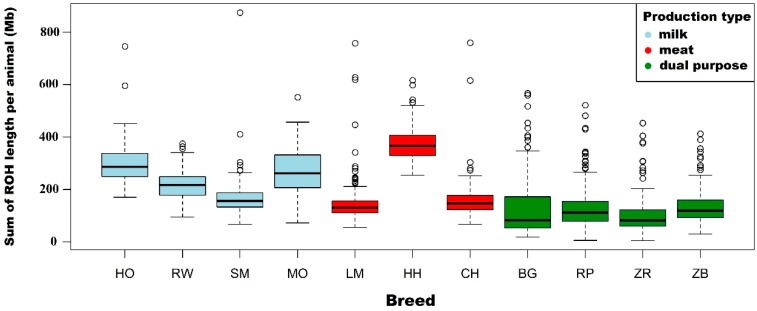
The average sum of ROH lengths per animal with division into breeds and cattle production types.

The highest sum of ROH lengths was observed for the Simmental individual (874 Mb). Animals with a high total length of ROH were also observed in the following breeds: Charolais (759 Mb), Limousin (757.9 Mb) and the Holstein-Friesian Black-and-White variety (745 Mb). Basic statistics on the length and number of ROH are described in Table 2 and are shown in Figure 1 and Figure 2.

### 3.2. Analysis of the F_ROH_ Inbreeding Coefficient

One of the most reliable inbreeding coefficients describing the phenomenon of inbreeding resulting from both past and recent relationship is F_ROH_ coefficient calculated for all identified ROHs, i.e., those with a length above 1 Mb.

On the one hand, the highest mean F_ROH_ values calculated for ROH above 1 Mb were observed for the Hereford breed (0.151), while intermediate values were identified for the Holstein-Friesian Black-and-White varieties (0.118), Montbeliarde (0.108) and Holstein-Friesian Red-and-White variety (0.087). On the other hand, relatively low F_ROH_ values were characteristic for breeds: Limousin (0.059), Charolais (0.065), Simmental (0.068) and conserved breeds: White-Backed (0.051), Polish Red (0.053), Polish Red-and-White (0.042) and Polish Black-and-White (0.054).

The highest mean F_ROH_ values calculated for ROH > 4 Mb were observed for the Hereford (0.101) breed. Meanwhile, intermediate values were characteristic for the following breeds: Holstein-Friesian Black-and-White variety (0.088), Montbeliarde (0.082) and Holstein-Friesian Red-and-White variety (0.060). The remaining breeds (SM, LM, CH, BG, RP, ZR and ZB) were characterized by low inbreeding coefficients in the range of 0.025–0.40.

**Figure 3:**
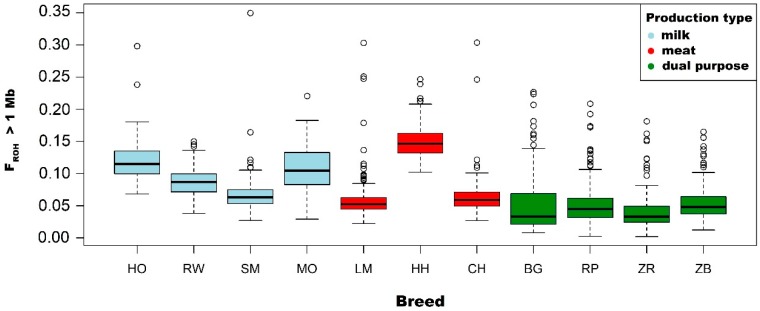
Values of F_ROH_ coefficients calculated for all identified ROH (>1 Mb) including division into breeds and cattle production types.

However, for F_ROH_ calculated on the basis of ROH with lengths above 8 and >16 Mb, the highest values were observed in the Montbeliarde breed (0.063 and 0.038). Intermediate values were characteristic for the Holstein-Friesian Black-and-White variety (0.061 and 0.029), Hereford (0.054 and 0.023) and the Holstein-Friesian Red-and-White variety (0.038 and 0.017) cattle. The remaining breeds and conserved breeds were characterized by lower values ranging from 0.019 to 0.028 for F_ROH_ above 8 Mb and 0.009–0.017 for F_ROH_ above 16 Mb. Basic statistics on the inbreeding coefficient calculated on the basis of ROH are described in Table 3 and are shown in Figure 3.

### 3.3. Characteristics of Genomic Regions with a High Frequency of ROH Occurrence

**Figure 4:**
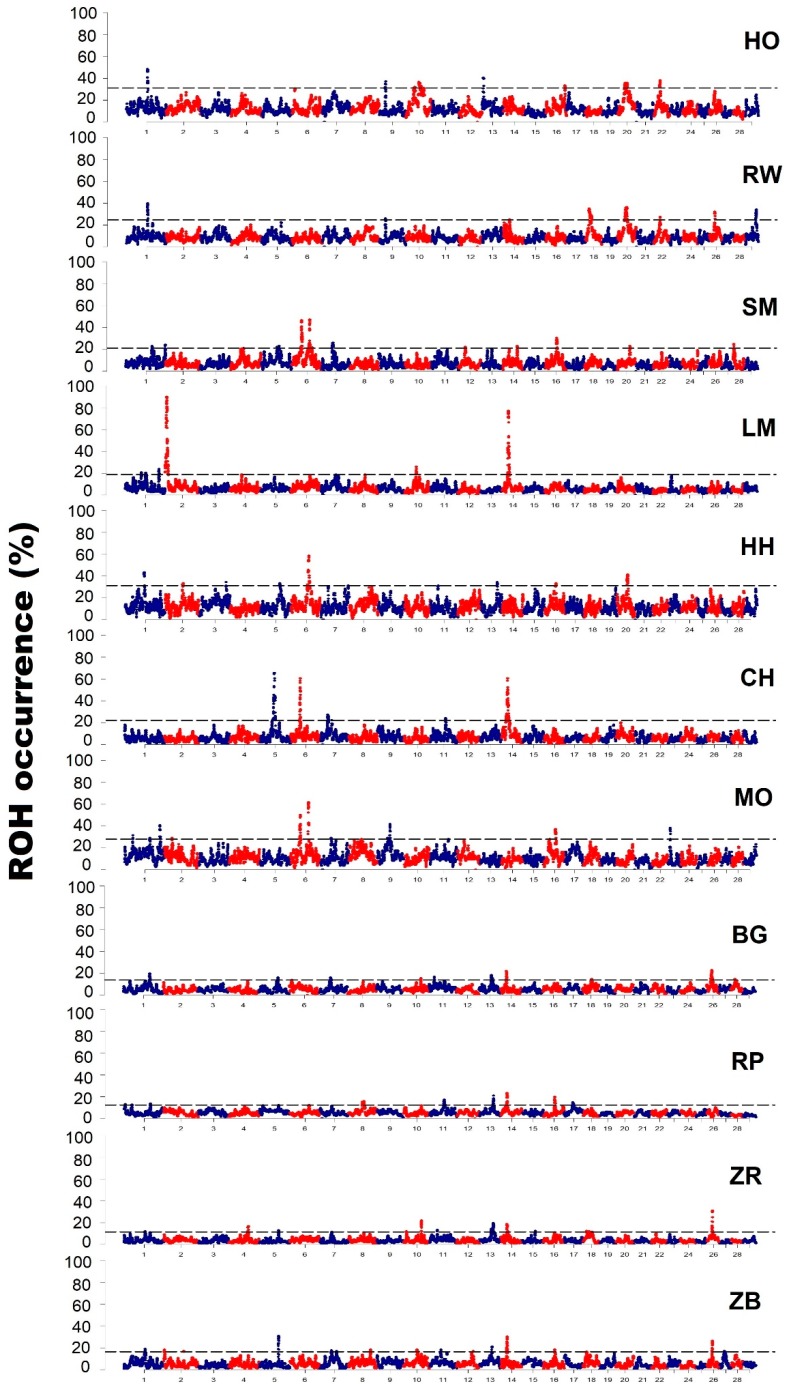
The frequency (%) at which individual genotype-based microarrays (SNPs) were observed in ROH for the individual cattle breeds (ROH patterns).

In order to identify genomic regions with the highest frequency of ROH and thus potentially under the influence of selection, ROH was analyzed on all 29 autosomes of 11 breeds of cattle maintained in Poland. Next, we calculated the frequency of individual SNPs that were present in ROH and selected the top 1% of the markers most commonly occurring in ROH. The neighboring markers with the highest frequency of ROH occurrence pointed to regions of the genome in which there are likely to be haplotypes that are a subject of selective pressure. The achieved results are presented in detail in Table 4 and shown schematically in Figure 4.

The analyzes allowed for the identification of 5 to 17 separate regions of the genome with a high frequency of ROH occurrence for particular breeds. The highest number of such regions was detected in the Polish Black-and-White breed (17) and the lowest in the Charolais breed (5). These regions were located on 5 (Charolaise) to 14 (Polish Red-and-White) autosomal chromosomes and had the length in range of 77.6 kb (Polish Red-and-White, three consecutive SNPs) to 1258.3 kb (Limousin; 206 consecutive SNPs). The average length of the region calculated for all breeds was 24,111.2 kb, while the average number of SNP per region was 38.5.

**Figure 5:**
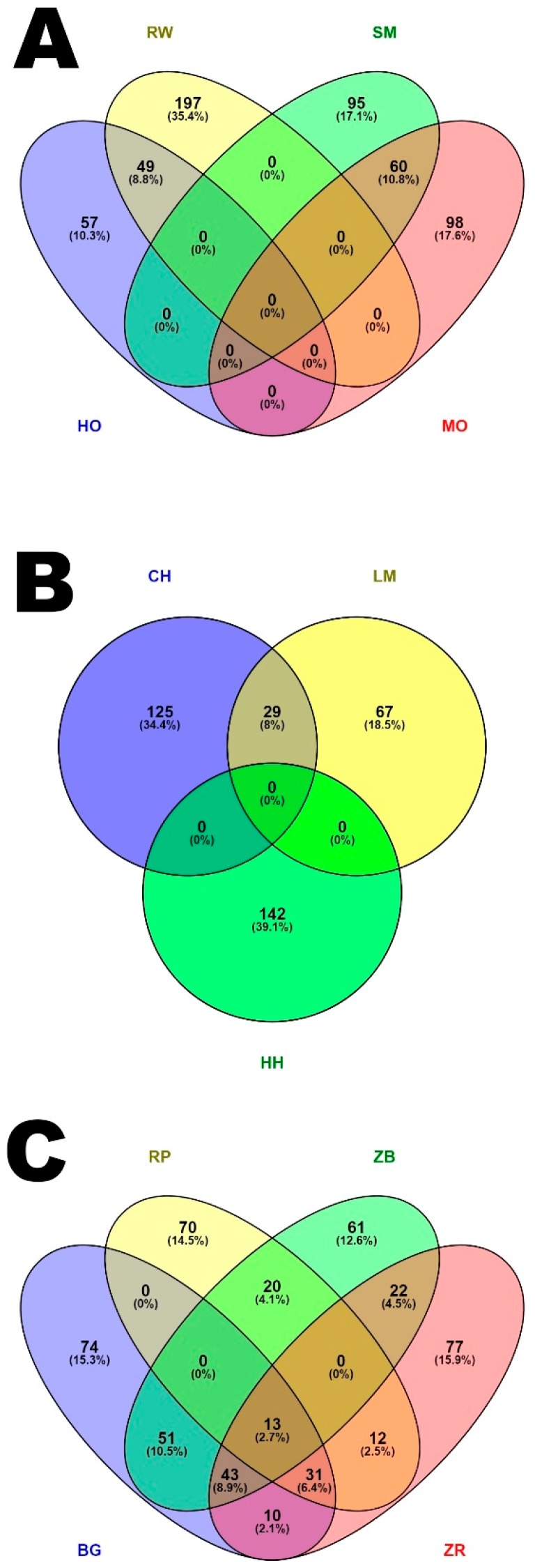
Venn diagram showing the number of genes located within ROH islands and detected between studied cattle breeds with division into breeds and cattle production types. (**A**) milk production type, (**B**) meat production type, (**C**) dual purpose production type.

In the identified regions with a high ROH occurrence, from 98 (LM) to 250 (RW) genes were detected depending on the breed. The number of genes located within ROH islands and detected in multiple breeds is shown in Figure 5 and the list of genes is presented in Supplementary Material S2.

The highest number of genes located within ROH islands and detected in multiple breeds was observed for MO and SM (60), HO and RW (49) as well as BG and ZB (51) breeds. Within dairy and meat production types (Figure 5B), no such genes for all breeds were observed, while genes located within ROH islands and detected in multiple breeds with the most similar constitutional type (HO and RW, MO and SM) were observed. In the case of conserved breeds (Figure 5C), there were 13 such genes between the four studied cattle breeds.

Genes located in regions with the highest frequency of ROH in individual populations were associated with numerous biological processes. The results of gene classification without enrichment analysis of individual processes are presented in Table 5. In the studied breeds, the largest number of genes were involved in cellular processes (from 42 to 109 genes) and metabolic processes (from 33 to 76 genes). Characteristic for conserved breeds, compared to other breeds, was a higher proportion of genes involved in processes related to the functioning of the immune system, reproduction, cell regulation and the organization of cellular components or biogenesis.

Among the identified genes, a significant part were involved in the pathways presented in Table 6. In most breeds, genes associated with the pathways responsible for integrin signaling were identified. Characteristic pathways for dairy breeds were those associated with angiogenesis and signaling of thyrotropin and gonadotropin releasing receptor. In the case of conserved dual-purpose breeds, pathways characterized by the highest number of identified genes were associated with the activation of B and T cells and inflammatory processes involving the cytokine and chemokine signaling pathway.

Within the regions of the genome with a high frequency of ROH occurrence, a number of genes with a confirmed influence on the level of production features have also been identified, including *DGAT1*, *MSTN*, *FABP4*, *ERBB3I*, *STAT1*, *GHR*, *SUFU*, *BTRC* or *CHUK*.

## 4. Discussion

### 4.1. Characteristics of Runs of Homozygosity

The characteristics of the ROH detected in this study corresponds well to the results obtained by other authors. Purfield et al. [1] identified ROH for various breeds of cattle, including European breeds: Holstein-Friesian, Limousin, Simmental, Hereford and Charolais. The authors showed that the average sum of ROH length for segments longer than 5 Mb and identified using BovineHD BeadChip microarrays was the highest for the Hereford (145 Mb) and Holstein-Friesian (115 Mb) breeds. However, the Limousin, Simmental and Charolais breeds were characterized by much lower values, respectively 45 Mb, 55 Mb and 50 Mb. In the case of all ROH (>1 Mb), the average sum of the ROH lengths was respectively higher: Hereford—245 Mb; Holstein-Frisian—195 Mb; Limousin—68 Mb; Simmental—85 Mb; and Charolais—85 Mb. Similar results were also obtained by Iacolina et al. [23], who compared European cattle breeds to the European bison using Illumina BovineHD BeadChip microarrays. The authors observed the highest average ROH lengths for Angus and Hereford breeds for the 1–5 Mb category (approximately 200 Mb). In the case of the Holstein-Friesian, Charolais, Simmental and Limousin breeds, these values were lower—in the range of approximately 100 Mb. In turn, in the research of Peripolli et al. [24] performed on Gyr cattle (Bos indicus), it was shown that the average sum of ROH lengths was in the range of 100 Mb per animal and the longest region was 108.9 Mb. These results are comparable with the results described in the present study for ROH with lengths >1 Mb and >4 Mb. In this study, the highest average ROH length was observed for the Hereford breed (378 Mb for ROH above 1 Mb and 253.4 Mb above 4 Mb), followed by the Holstein-Friesian Black-and-White variety (295.1 and 220.8 MB), and later, respectively, the following breeds: Charolais (163.2 Mb and 97.2 Mb), Simmental (169.1 and 87.4 Mb) and Limousin (147.4 Mb for ROH above 1 Mb and 80.3 Mb above 4 Mb). Apart from analyzing the ROH of high-production cattle breeds kept in Poland (HO, RW, SM, LM, MO, CH and HH), which are largely genetically close to their European counterparts, Polish indigenous breeds of cattle were also examined (BG, RP, ZR and ZB). These breeds are included in the genetic resources conservation programs. An analysis of ROH showed notable differences in the length and amount of ROH between native breeds and production breeds. Regarding the average number of ROH per animal for native breeds, it was shown to be in the range from 21 to 30, while for production breeds, it was in the range from 39 to 81. A similar trend can be observed for the average sum of ROH lengths—in the case of native breeds, it was lower and ranged from 105 to 135 Mb, while in the case of production breeds, it was in the range from 147 to 378 Mb.

According to the results obtained, we hypothesized that much higher mean length values and ROH numbers obtained for highly selected breeds in comparison to native breeds may be associated with strong directional selection and widespread and intensive use of artificial insemination. It has been proven that the use of biotechnics and intensive artificial selection significantly reduce genetic variability and increase the degree of relationship between animals [25]. In addition, native breeds were characterized by low, compared to commercial breeds, average amounts of ROH with a length of more than 8 Mb (2.2–3.8 in the case of conserved breeds and 3.1–10.1 in production breeds). Due to the fact that the occurrence of long segments of ROH is related to the recent relationship within the parental population [26], small amounts of long ROH segments indicate a low degree of the close relationship between animals from conserved herds. Similar differences between native breeds and production breeds were also observed by other authors [1]. The implementation of the genetic resources conservation program itself (Program for the Conservation of Genetic Resources of Farm Animals), which focuses on minimizing inbreeding within protected populations, seems to be of great importance for the obtained results. Not without significance is the admission in the protected populations of a certain share of the blood of other breeds, which increases the genetic variation and diversity of segregating haplotypes.

Differences in the ROH statistics between highly selected and native breeds may also result from the intensity of selection used within these populations. Kim et al. [27] studied Holstein cattle from populations with varying intensity of selection and observed that in animals undergoing intensive selection, the average length of ROH per animal was about 6.67 Mb, while for extensively selected animals, it was 6.26 Mb. In the case of an average amount of ROH per animal, the values were as follows: 40.4 for intensively selected animals and 31.1 for extensively selected animals. The same trend can be seen in the results of the present research by comparing intensively the selected production breeds and native breeds covered by the genetic resources conservation program.

### 4.2. Runs of Homozygosity as a Tool of Inbreeding Estimation 

F_ROH_ (inbreeding coefficient calculated on the basis of ROH) is widely regarded as a reliable measure of individual autozygosity and provides information on the degree of inbreeding of individual herds of animals, taking into account both past and recent relatedness of individuals [3,28]. The literature data also indicated that F_ROH_ may be a better estimator of individual autozygosity than those based on pedigree data (F_PED_) [28]. However, it should be noted that the use of medium density SNP arrays, such as the one used in this research, leads to limited identification of short ROH that contribute significantly to ancient inbreeding [17].

In many studies on cattle, a strong or moderate correlation between F_PED_ and F_ROH_ values was observed: 0.73 for ROH longer than 1 Mb and 0.70 for ROH longer than 10 Mb [1] and from 0.485 to 0.715 for ROH longer than 1 Mb [28]. In our previous study [29] performed on Polish Holstein-Friesian cattle, slightly lower values of the correlation coefficients between F_ROH_ and F_PED_ were observed (in the range from 0.308 to 0.505 for ROH with a length of more than 1 Mb). This is mainly the result of using Spearman’s rank correlation coefficients, more appropriate for data sets with a large number of outliers and those not showing a normal distribution [30], but often giving lower correlation values.

It is worth mentioning that the correlation between F_ROH_ and F_PED_ coefficients rises with the increase in the length of ROH segments used for calculations. According to Marras et al. [26], this is due to the fact that ROH reflects both past and recent animal relatedness, while F_PED_ coefficients are based on pedigree records that may not contain information from many past generations. Therefore, F_ROH_ coefficient calculated on the basis of long ROH better reflects the recent relationship, and the F_ROH_–F_PED_ correlation is usually higher considering only long ROH segments in the calculation. In studies conducted on pigs, Saura et al. [31] showed that the mean value of F_ROH_ coefficient calculated for ROH with lengths >5 Mb was close to F_PED_, while the mean for F_ROH_ calculated for ROH longer than 5 Mb was about four times lower than the average F_PED_. Another study was presented by Scraggs et al. [32], which suggested that F_PED_ does not determine true kinship within the Wagyu cattle population, as there were clear differences between F_ROH_ and F_PED_ coefficients. The authors showed much lower values of F_PED_ coefficients compared to F_ROH_. These results are consistent with data obtained by other authors for cattle [26,33] and pigs [31], in which the F_ROH_ coefficient was characterized by higher values than F_PED_, suggesting that F_PED_ may underestimate the degree of inbreeding of the studied populations.

Due to the information presented above, in this research, thes F_ROH_ coefficient was used to assess the level of inbreeding of the selected cattle population maintained in Poland. The highest mean values of inbreeding coefficient calculated for ROH with a length above 1 Mb were observed in the Hereford (0.151), Holstein-Friesian Black-and-White variety (0.118) and Montbeliarde (0.108). The lowest F_ROH_ values, as expected, were noted for native breeds: White-Backed (0.051), Polish Red (0.053), Polish Red-and-White (0.042) and Polish Black-and-White (0.054). In addition, mean F_ROH_ coefficients calculated for ROH longer than 8 and 16 Mb were the highest in Montbeliarde, Holstein-Friesian Black-and-White and Hereford breeds, which suggests a relatively high degree of relatedness between animals and a reduction in genetic variation.

The inbreeding level estimated in this work for individual cattle breeds generally corresponds to the results obtained by other authors. In the study of Ferenčaković et al. [28], F_ROH_ inbreeding coefficients for ROH with lengths above 1 Mb was calculated and high average autozygosity levels (0.151) were found for strongly selected Brown Swiss cattle population and significantly lower for native cattle (0.052 for Pinzgauer cattle and 0.066 for Tyrol Gray). In addition, in another study, Ferenčaković et al. [20] observed varying F_ROH_ values, depending on the cattle breed: 0.156 for brown Swiss cattle; 0.088 for Fleckvieh cattle; 0.099 for Norwegian Red cattle; 0.087 for Tyrol Gray cattle and 0.09 for Simmental cattle. In the case of Holstein cattle maintained in the USA, F_ROH_ calculated for ROH with a length of more than 5 Mb was low and amounted to 0.038 [34]. In Holstein cattle kept in Europe, higher F_ROH_ coefficients were found in the range from 0.081 for ROH > 1 Mb to 0.046 for ROH > 5 Mb [1]. F_ROH_ values for the Polish Holstein-Friesian cattle described in this paper was slightly higher than that observed for other populations of Holstein-Friesian cattle in Europe; however, similar to the other high-production European dairy breeds [1].

### 4.3. Analysis of Genomic Regions with a High Frequency of ROH Occurrence 

The examination of runs of homozygosity distribution across the genome shows that their dispersal in particular regions of the genome is unique and they can form characteristic patterns depending on a given population. These genomic regions with the highest frequency of ROH occurrence were called by Nothnagel et al. [13] and Pemberton et al. [14] with a term “ROH islands”. In studies of many authors, it is suggested that these regions are shaped by the influence of strong selection pressure on variants located at a given locus [10,11,12,14]. In addition, Zhang et al. [25] confirmed a significant correlation between short regions with a high frequency of occurrence of ROH and genomic regions considered to be under the influence of selection using methods based on F_ST_ coefficient and iHS (integrated haplotype score).

An overview of the available literature indicates that many authors have observed and described the occurrence of ROH islands and their relationship with QTL for important production traits in cattle. Purfield et al. [1] observed that the genomic regions located on BTA 7, BTA 14, BTA 16 and BTA 18 were characterized by a high frequency of ROH occurrence and included important genes related to immune traits, muscularity and ease of calving. In particular, BTA 5 and BTA 9 were characterized by an increased number of long ROH above 20 Mb, within which there were numerous QTL regions associated with the production of milk fat and growth characteristics of cattle [1].

In this study, several genomic regions with extremely high levels of autozygosity and frequency of ROH were observed in individual breeds of cattle. An example of such a region may be the initial sequence of chromosome 2 in the Limousin breed, within which there is a myostatin gene locus (*MSTN*), which is a strong QTL for muscle traits in this breed [35]. A similar region on BTA 2 was found by Marras et al. [26] by studying Piedmontese cattle, where almost 90% of all animals were characterized by the occurrence of ROH in the proximal part of the second chromosome. Other regions of interest, potentially influenced by the directional selection, may be two regions located on chromosome 5 and 6 in the Charolais breed, within which genes related to growth factors and coat color are located. One of these genes is *ERBB3*—encoding the epidermal growth factor receptor. The literature showed that *ERRB3* controls the proliferation and myogenic diversity of muscle stem cells [36]. Then, there are the *INHBC* and *INHBE* genes that are part of the TGFB signaling pathway—a transforming growth factor beta, which is an essential regulator of fibroblast proliferation, collagen synthesis in muscle tendons and in the case of muscular tissue can affect muscle atrophy [37]. Therefore, these genes may potentially be associated with meat traits of Charolais cattle that are subject to selection in this breed. Moreover, in various studies [38] within this ROH island, the *PMEL* gene was proposed as the one associated with the white coat color of Charolais cattle. Another region with high homozygosity was observed in the proximal part of chromosome 14. This region was characterized by a high frequency of occurrence of ROH, in the range of 60% to 80% in the population of LM and CH breeds (respectively); however, elevated levels of homozygosity were also observed for this region in other breeds of cattle: RW, BG, RP, ZB and ZR. The identified region included the *DGAT1* gene locus, with known influence on fat percentage in milk and *FABP4* gene associated in numerous previous studies with transport, regulation and lipid metabolism [39]. In addition, the *FABP4* gene was presented as affecting the qualitative characteristics of meat in cattle and also affecting fat content in meat [40]. In turn, in the studies of Zhou et al. [41], the effect of the *FABP4* gene on the amount of milk produced and protein content in milk was also observed.

When comparing the results of this research to literature data, a large similarity of the location of ROH islands identified in different breeds of cattle can be observed. In studies of Mészáros et al. [42], a ROH island was observed in Tyrol Gray cattle located on chromosome 6 in the 36–41 Mb position. In the same chromosomal region, in this study, regions with an increased incidence of ROH were identified for the Montbeliarde, Simmental and Charolais breeds. The research performed on domestic cattle allowed the detection of four ROH islands, two of which were also identified in this study: the first on BTA 6 in the location of 38.2–39.4 Mb, which coincides with the ROH islands observed in the MO, SM and CH breeds; the second on BTA 16 with the location of 43.8–45.0 Mb, which coincides with the regions with high ROH frequency for the MO, SM, HH, RP and ZB breeds [43]. In the studies of Marras et al. [26], the authors observed characteristic ROH islands on chromosomes 2 and 6. The ROH island on chromosome 2 was characteristic to Piemontese cattle and was associated with the MSTN gene. A similar effect resulting from strong selection pressure on the *MSTN* gene was observed in this research in Limousin cattle. In contrast, ROH island located on chromosome 6 in Holstein and Italian Brown dairy cattle included *ABCG2* and *FAM13A1* genes, which are known to be associated with milk traits [44,45]. The same genes were identified in regions with a high incidence of ROH in this study in the following cattle breeds: SM, CH, MO (*ABCG2* gene) and HO, RW, SM, CH, MO and ZB (*FAM13A1* gene).

In the present study, in regions with a high frequency of ROH occurrence, a number of other genes associated with cattle production traits were also observed. An example of such a gene identified in the ROH island on chromosome 2 is the *STAT1* gene observed in ZB cattle. Cobanoglu et al. [46]’s study demonstrated the important role of this gene in the regulation process of transcription of other genes involved in the synthesis of milk proteins and fat metabolism. In dairy cattle breeds HO and RW, within ROH islands, the *GHR* gene was observed, which has a significant impact on the quantity and composition of milk [47,48]. In addition, the same breeds have demonstrated the potential effect of selection pressure on the *TRH* gene belonging to the thyrotropin secretion signaling pathway, which has a significant effect on growth hormone secretion and prolactin [49]. Literature data confirm that exogenous stimulation of these hormones promotes a significant increase in milk yield [50,51]. Further examples of genes identified in ROH islands in the RW, BG, ZR and ZB breeds are SUFU and BTRC, that regulate the proliferation of lactate stem cells. In humans, the *SUFU* gene acts as a negative regulator of the hedgehog signaling pathway, whereas the *BTRC* gene controls the self-renewal process of the mammary gland stem cells [52]. In BG, ZR and ZB breeds, in the ROH islands, we also detected the *CHUK* gene, which, in the literature, is described as one of the genes responsible for the development of mammary gland and lactation [53]. In addition, genes participating in the Wnt signaling pathway were identified in RW, SM, BG and RP cattle breeds. The Wnt signaling pathway is responsible for the regulation of cells proliferation, their morphology, apoptosis processes and differentiation as well as the development of the mammary gland [53]. Another group of genes identified in the ROH islands are genes related to the signaling pathway of the angiogenesis process observed in the following breeds: HO, SM, MO, RP and HH, which may also be associated with the development of the mammary gland and affect its blood supply [54]. In addition, in the case of conserved breeds, the highest number of genes identified in the ROH islands belonged to the metabolic pathways associated with the activation of B, T cells and inflammatory processes involving cytokine and the chemokine signaling pathway, which may be related to the higher resistance to diseases characteristic of these breeds.

When analyzing genes occurring in ROH islands and observed in more than one breed, 13 genes were found between BG, RP, ZB and ZR cattle breeds. These genes have been described previously as affecting growth in cattle and humans (*TGS1*, *LYN*, *CHCHD7*, *SDR16C5*, *TMEM68*) [55,56,57,58], ease of calving (*RPS20*) [59] and traits related to puberty (*FAM110B*) [60]. These traits are also subject of selection in cattle of different breeds and may have been fixed during a long-term breeding process.

## 5. Conclusions

To conclude, in the present study we examined the characteristics of ROH identified on the basis of genomes of 11 cattle breeds. The whole genome approach allowed the presentation of visible differences in the length, quantity and frequency of ROH between the analyzed breeds. These differences were particularly pronounced between commercial and conserved breeds. Moreover, we calculated the levels of genomic inbreeding within individual cattle breeds, which showed a higher level of inbreeding characteristic to commercial breeds, especially meat breeds. In addition, regions of the genome that were most commonly associated with ROH were identified, which may reveal signatures of recent selection and describe ROH patterns of the analysed cattle breeds. Within these genomic regions, a number of genes with a confirmed influence on the level of production traits were observed, including *DGAT1*, *MSTN*, *FABP4*, *ERBB3*, *STAT1*, *GHR*, *SUFU*, *BTRC* or *CHUK*. The presented results and identified genes can be a basis to a further research focused on the identification of genes and markers essential in determining the most important production traits in cattle.

## Figures and Tables

**Table 1:** Numbers and production types of the studied cattle breeds.

Breed	Number of Individuals	Production Type *
Holstein-Friesian Black-and-White variety (HO)	299	M
Holstein-Friesian Red-and-White variety (RW)	231	M
Simmental (SM)	161	M
Limusin (LM)	197	MS
Hereford (HH)	77	MS
Charolais (CH)	99	MS
Montbeliarde (MO)	86	M
White-Backed (BG)	136	D
Polish Red (RP)	283	D
Polish Red-and-White (ZR)	210	D
Polish black and white (ZB)	152	D

* M—milk, MS—meat, D—dual purpose.

**Table 2:** Number and length of ROH in selected length categories.

Breed		Statistics	ROH Length Category (Mb)				
			1+	2+	4+	8+	16+
Holstein-Friesian Black-and-White variety (*n* = 299)	Number of ROH per animal	Mean	53.3	42.3	22.4	10.1	3.0
		SD	7.3	6.8	5.2	3.4	1.9
		Min	36	26	9	2	0
		Max	79	70	52	32	12
	Length of ROH per animal (Mb)	Mean	295.1	277.0	220.8	151.3	74.2
		SD	68.5	68.6	67.6	63.1	52.4
		Min	170.4	151.0	77.8	25.3	0
		Max	745.0	729.2	678.7	557.0	402.0
Holstein-Friesian Red-and-White variety (*n* = 231)	Number of ROH per animal	Mean	44.3	33.8	16.5	6.7	1.9
		SD	7.0	6.6	4.9	2.9	1.5
		Min	24	18	6	1	0
		Max	65	57	35	17	8
	Length of ROH per animal (Mb)	Mean	216.7	199.4	150.7	96.0	43.0
		SD	53.4	53.9	52.5	45.0	34.3
		Min	95.3	82.8	41.5	13.3	0
		Max	375.1	361.5	316.6	225.6	185.6
Simmental (*n* = 161)	Number of ROH per animal	Mean	46.1	32.0	10.7	3.1	0.8
		SD	8.3	7.4	4.4	2.9	1.7
		Min	23	15	2	0	0
		Max	67	57	32	26	18
	Length of ROH per animal (Mb)	Mean	169.1	146.0	87.4	50.0	22.0
		SD	74.8	75.3	71.9	69.7	56.9
		Min	68.0	49.6	9.2	0	0
		Max	874.1	859.0	787.2	755.2	661.3
Limousin (*n* = 197)	Number of ROH per animal	Mean	38.5	26.2	9.2	3.1	1.0
		SD	6.9	6.0	4.6	3.2	2.1
		Min	18	13	1	0	0
		Max	62	50	35	28	17
	Length of ROH per animal (Mb)	Mean	147.4	127.1	80.3	47.5	27.8
		SD	80.5	80.1	80.3	76.4	68.6
		Min	56.0	48.1	4.4	0	0
		Max	757.9	746.0	709.9	670.2	556.6
Hereford (*n* = 77)	Number of ROH per animal	Mean	80.6	66.5	31.4	9.6	2.3
		SD	7.5	7.0	5.7	4.0	2.0
		Min	62	47	19	4	0
		Max	98	84	51	25	8
	Length of ROH per animal (Mb)	Mean	378.0	354.5	253.4	134.0	56.4
		SD	73.9	74.4	75.7	71.6	53.3
		Min	255.0	228.4	135.8	40.5	0
		Max	616.5	593.1	509.3	376.9	228.3
Charolais (*n* = 99)	Number of ROH per animal	Mean	40.0	29.2	11.9	3.6	0.8
		SD	7.7	6.6	5.0	3.5	1.9
		Min	23	15	3	0	0
		Max	59	49	40	23	13
	Length of ROH per animal (Mb)	Mean	163.2	145.6	97.2	52.5	21.8
		SD	87.4	87.1	88.2	84.4	68.7
		Min	66.9	50.6	19.4	0	0
		Max	759.0	737.4	699.0	662.2	555.0
Montbeliarde (*n* = 86)	Number of ROH per animal	Mean	45.9	34.2	17.6	9.2	3.6
		SD	7.5	6.8	5.4	4.0	2.2
		Min	28	16	4	0	0
		Max	69	51	33	20	12
	Length of ROH per animal (Mb)	Mean	269.8	251.0	204.7	157.6	94.8
		SD	85.4	86.6	86.4	80.6	65.2
		Min	73.2	54.1	21.3	0	0
		Max	551.6	540.2	506.5	453.0	345.4
White-Backed (*n* = 136)	Number of ROH per animal	Mean	23.9	17.1	8.5	3.8	1.5
		SD	8.9	8.4	6.9	4.5	2.6
		Min	7	5	1	0	0
		Max	46	42	31	21	12
	Length of ROH per animal (Mb)	Mean	127.6	116.6	93.0	67.4	42.2
		SD	113.4	113.4	111.2	100.0	80.4
		Min	19.3	15.1	4.4	0	0
		Max	566.6	559.9	529.4	477.4	391.3
Polish Red (*n* = 283)	Number of ROH per animal	Mean	23.3	17.7	9.5	4.3	1.3
		SD	9.4	8.4	5.3	3.2	1.5
		Min	3	1	0	0	0
		Max	70	64	34	18	8
	Length of ROH per animal (Mb)	Mean	131.7	122.3	98.8	69.0	35.1
		SD	86.1	84.8	76.1	64.9	48.9
		Min	6.0	2.4	0	0	0
		Max	521.6	511.6	454.7	382.1	298.7
Polish Red-and-White (*n* = 210)	Number of ROH per animal	Mean	21.8	14.4	5.1	2.2	0.6
		SD	7.2	5.8	3.6	2.5	1.4
		Min	3	0	0	0	0
		Max	41	32	18	13	9
	Length of ROH per animal (Mb)	Mean	105.7	91.2	61.3	38.4	18.7
		SD	74.6	71.2	65.4	57.6	46.0
		Min	5.0	0	0	0	0
		Max	453.1	436.0	399.2	358.3	336.3
Polish Black-and-White (*n* = 152)	Number of ROH per animal	Mean	30.8	21.8	9.0	3.6	1.0
		SD	9.5	7.6	4.9	3.0	1.6
		Min	10	6	0	0	0
		Max	68	41	23	13	8
	Length of ROH per animal (Mb)	Mean	135.8	120.7	84.9	54.4	25.0
		SD	71.1	69.4	66.7	58.7	48.3
		Min	31.2	20.3	0	0	0
		Max	412.5	401.0	377.7	342.5	277.5

**Table 3:** Values of F_ROH_ coefficients in selected ROH length categories.

Breed	Statistics	ROH Length Category (Mb)				
		1+	2+	4+	8+	16+
Holstein-Friesian Black-and-White variety	Mean	0.118	0.111	0.088	0.061	0.029
	SD	0.027	0.027	0.027	0.025	0.021
	Min	0.068	0.060	0.031	0.010	0.000
	Max	0.298	0.292	0.271	0.223	0.161
Holstein-Friesian Red-and-White variety	Mean	0.087	0.080	0.060	0.038	0.017
	SD	0.021	0.022	0.021	0.018	0.014
	Min	0.038	0.033	0.017	0.005	0.000
	Max	0.150	0.145	0.127	0.090	0.074
Simmental	Mean	0.068	0.068	0.035	0.019	0.009
	SD	0.030	0.030	0.029	0.028	0.023
	Min	0.027	0.020	0.004	0.000	0.000
	Max	0.350	0.344	0.315	0.302	0.265
Limousin	Mean	0.059	0.051	0.032	0.019	0.011
	SD	0.032	0.032	0.032	0.031	0.027
	Min	0.022	0.019	0.002	0.000	0.000
	Max	0.303	0.298	0.284	0.268	0.223
Hereford	Mean	0.151	0.142	0.101	0.054	0.023
	SD	0.029	0.029	0.030	0.029	0.021
	Min	0.102	0.091	0.054	0.016	0.000
	Max	0.247	0.237	0.204	0.151	0.091
Charolais	Mean	0.065	0.058	0.039	0.021	0.009
	SD	0.035	0.035	0.035	0.033	0.027
	Min	0.027	0.020	0.007	0.000	0.000
	Max	0.304	0.295	0.280	0.265	0.222
Montbeliarde	Mean	0.108	0.100	0.082	0.063	0.038
	SD	0.034	0.034	0.035	0.032	0.026
	Min	0.029	0.022	0.009	0.000	0.000
	Max	0.221	0.216	0.203	0.181	0.138
White-Backed	Mean	0.051	0.047	0.037	0.027	0.017
	SD	0.045	0.045	0.044	0.040	0.032
	Min	0.008	0.006	0.002	0.000	0.000
	Max	0.227	0.224	0.212	0.191	0.157
Polish Red	Mean	0.053	0.049	0.040	0.028	0.014
	SD	0.034	0.034	0.030	0.026	0.019
	Min	0.002	0.001	0.000	0.000	0.000
	Max	0.209	0.205	0.182	0.153	0.120
Polish Red-and-White	Mean	0.042	0.036	0.025	0.015	0.007
	SD	0.030	0.029	0.026	0.023	0.018
	Min	0.002	0.000	0.000	0.000	0.000
	Max	0.181	0.174	0.160	0.143	0.135
Polish Black-and-White	Mean	0.054	0.048	0.034	0.022	0.010
	SD	0.028	0.028	0.027	0.023	0.019
	Min	0.012	0.008	0.000	0.000	0.000
	Max	0.165	0.160	0.151	0.137	0.111

**Table 4:** Characterization of genomic regions with the highest frequency of ROH occurrence.

Breed	Chromosome	Start (bp)	End (bp)	Number of SNPs	Length of the Region (kb)
Holstein-Friesian Black-and-White variety	1	82,928,948	85,264,656	46	2365
	9	24,369,582	25,408,468	20	1038
	10	36,486,868	36,807,581	6	320
	10	52,190,618	61,064,570	128	8873
	13	4,755,215	6,822,805	40	2067
	16	74,532,751	75,892,097	28	1359
	20	25,850,728	32,074,342	73	6223
	20	34,728,244	36,917,645	41	2189
	22	21,266,612	23,914,818	44	2648
Holstein-Friesian Red-and-White variety	1	82,951,366	85,525,315	46	2573
	9	24,369,582	25,359,074	19	989
	14	25,254,540	25,638,580	6	384
	18	13,901,770	18,449,746	77	4547
	18	23,196,347	25,471,374	44	2275
	20	24,544,146	36,311,419	149	11,767
	22	23,365,188	23,914,818	12	549
	26	21,180,893	23,071,595	35	1890
	29	40,858,913	440,85,769	57	3226
Simmental	1	103,675,933	105,570,832	31	1894
	1	155,955,828	156,710,174	14	754
	4	50,547,931	51,370,504	6	822
	5	63,555,403	64,190,317	9	634
	5	70,338,965	71,546,802	18	1207
	6	37,252,345	42,714,287	132	5461
	6	65,217,698	65,978,639	8	760
	6	67,752,994	73,254,801	78	5501
	6	81,499,583	82,047,313	7	547
	7	41,565,963	46,354,401	72	4788
	12	26,967,177	27,337,843	9	370
	14	57,672,324	58,740,723	16	1068
	16	42,892,437	45,552,538	29	2660
	16	46,069,918	47,201,903	18	1131
	20	48,114,351	50,086,666	35	1972
	28	2,181,928	2,924,302	5	742
Limousin	1	63,421,529	64,276,370	14	854
	1	80,548,510	81,656,974	12	1108
	1	1.33 × 10^8^	1.35 × 10^8^	26	1597
	2	35,126	12,632,490	206	12,597
	4	45,577,225	46,381,877	6	804
	7	58,809,602	58,923,345	4	113
	10	45,864,066	47,664,187	36	1800
	14	22,643,306	29,543,761	134	6900
Hereford	1	73,757,146	76,938,175	47	3181
	2	68,877,969	72,583,890	32	3705
	3	1.05 × 10^8^	1.06 × 10^8^	14	521
	5	75,114,559	79,165,498	62	4050
	6	65,380,200	74,354,100	131	8973
	7	1.07 × 10^8^	1.08 × 10^8^	27	1237
	11	28,946,979	29,079,159	4	132
	13	64,253,779	65,817,864	21	1564
	16	43,371,269	45,376,614	25	2005
	20	39,538,676	44,414,152	96	4875
Charolais	5	53,263,967	62,180,846	94	8916
	6	36,708,862	40,063,618	72	3354
	7	28,182,762	31,973,748	50	3790
	11	60,738,925	61,700,872	11	961
	14	18,756,025	29,543,761	191	10787
Montbeliarde	1	32,509,969	33,036,107	11	526
	1	99,477,567	1.01 × 10^8^	29	1385
	1	1.39 × 10^8^	1.42 × 10^8^	41	2840
	2	29,055,572	29,627,722	16	572
	6	36,829,725	40,580,921	82	3751
	6	70,349,791	73,092,782	55	2742
	7	41,805,531	44,136,041	37	2330
	8	49,981,054	50,725,941	18	744
	9	40,287,003	44,951,803	42	4664
	9	46,351,157	50,728,426	67	4377
	11	72,069,940	72,864,887	12	794
	16	22,179,895	23,037,476	7	857
	16	43,424,406	47,558,131	50	4133
	23	9,020,371	10,665,897	34	1645
White-Backed	1	1.02 × 10^8^	1.06 × 10^8^	68	4203
	5	75,794,378	77,311,671	33	1517
	6	9,404,648	10,424,905	10	1020
	7	42,521,261	46,626,888	38	4105
	10	70,894,537	71,985,171	27	1090
	11	20,558,025	21,817,694	36	1259
	13	47,546,608	50,701,854	37	3155
	13	53,618,942	55,006,836	21	1387
	14	24,145,838	26,473,490	44	2327
	18	36,146,356	38,147,823	25	2001
	26	18,335,079	24,531,763	100	6196
	26	25,170,222	25,657,642	10	487
	28	16,727,989	17,304,235	14	576
Polish Red	1	4,648,383	5,351,369	12	702
	1	31,269,020	31,551,425	3	282
	1	1.04 × 10^8^	1.05 × 10^8^	13	1042
	8	55,145,132	57,339,395	39	2194
	8	58,789,069	60,589,007	34	1799
	8	61,536,940	63,000,189	32	1463
	8	63,162,363	63,901,386	16	739
	11	55,809,281	60,250,739	49	4441
	13	53,347,036	57,016,938	68	3669
	14	23,384,687	26,597,692	57	3213
	16	42,892,437	46,625,869	37	3733
	17	34,139,617	37,933,239	42	3793
Polish Red-and-White	1	83,838,758	83,916,372	3	77
	4	75,890,428	77,635,835	31	1745
	5	76,317,361	77,311,671	26	994
	10	11,707,725	12,020,216	6	312
	10	70,736,766	72,202,330	33	1465
	11	29,822,671	30,945,111	31	1122
	13	46,150,079	47,990,990	40	1840
	13	50,950,127	51,165,507	4	215
	13	52,837,622	56,190,025	57	3352
	14	24,275,232	28,332,133	45	4056
	15	52,311,393	52,910,307	14	598
	18	13,901,770	15,594,562	26	1692
	18	23,196,347	23,949,849	21	753
	26	19,727,292	23,461,479	63	3734
Polish Black-and-White	1	82,787,221	84,515,050	19	1727
	2	784,712	2,415,461	36	1630
	2	78,556,325	80,096,393	30	1540
	5	75,627,333	77,679,706	43	2052
	7	44,901,489	47,213,804	22	2312
	7	66,303,743	66,645,827	7	342
	8	87,308,122	88,974,063	26	1665
	10	52,606,823	54,514,075	44	1907
	10	56,464,919	56,812,824	3	347
	11	44,061,322	45,008,048	20	946
	12	65,092,442	65,481,742	6	389
	13	48,485,992	50,813,233	32	2327
	14	23,054,179	26,542,736	61	3488
	16	43,810,410	45,017,787	14	1207
	18	14,115,136	15,099,438	14	984
	26	20,365,711	23,129,849	45	2764
	27	17,593,646	18,991,970	27	1398

**Table 5:** Biological processes most often associated with genes identified within genome regions with a high frequency of ROH occurrence.

Biological Processes/Breeds	The Number of Genes Involved in the Process										
	HO	RW	SM	LM	HH	CH	MO	BG	RP	ZB	ZR
Organization of cellular components or biogenesis (GO: 0071840)	11	21	21	14	17	21	16	33	21	31	24
Cellular processes (GO: 0009987)	47	98	76	42	77	73	65	103	71	109	96
Location (GO: 0051179)	7	25	16	9	26	13	17	28	17	29	27
Reproduction (GO: 0000003)	1	1	2	1	3	0	3	1	4	5	4
Biological regulation (GO: 0065007)	15	28	19	15	29	22	20	32	25	33	31
Response to stimulus (GO: 0050896)	15	24	20	19	25	26	26	28	21	32	24
Development processes (GO: 0032502)	14	13	14	6	14	21	9	20	15	23	16
Processes of multicellular organisms (GO: 0032501)	9	15	13	6	18	15	9	14	15	20	15
Adhesion processes (GO:0022610)	3	2	3	1	3	3	0	3	2	3	4
Locomotion (GO:0040011)	2	3	4	1	3	2	1	4	1	4	1
Metabolic processes (GO:0008152)	41	76	58	33	48	60	55	84	47	83	74
Processes of the immune system (GO:0002376)	5	4	5	1	2	7	1	7	7	5	5

**Table 6:** Selected pathways associated with genes identified within the genome regions with the highest frequency of ROH occurrence.

Breed	Pathways	Genes
HO	Angiogenesis (P00005)	*EPHB3*, *DLL4*
	Integrin signal pathway (P00034)	*LAMB3*, *ITGA2*
	Thyrotropin-releasing hormone receptor signaling pathway (P04394)	*TRH*, *GNB5*
	CCKR signaling map (P06959)	*AP2M1*, *HDC*
	Gonadotropin-releasing hormone receptor pathway (P06664)	*ISL1*, *GNB5*
RW	Gonadotropin-releasing hormone receptor pathway (P06664)	*GNAO1*, *PRKAA1*, *FST*, *MAP4K2*, *ISL1*, *ESRRA*, *PTGER4*
	Inflammation involving chemokine and cytokine signaling pathway (P00031)	*GNG3*, *GNAO1*, *CCL22*, *NFKB2*, *ITGA2*, *PLCB3*
	Wnt signaling pathway (P00057)	*GNG3*, *CDH15*, *BTRC*, *PPP2R5B*, *SIAH1*, *PLCB3*
	Endogenous cannabinoid signaling pathway (P05730)	*GNG3*, *GNAO1*, *PLCB3*
	Thyrotropin-releasing hormone receptor signaling pathway (P04394)	*GNG3*, *TRH*, *PLCB3*
	CCKR signaling map (P06959)	*AP2M1*, *MEN1*, *BAD*
SM	Angiogenesis (P00005)	*KDR*, *PIK3CD*, *PDGFRA*
	Wnt signaling pathway (P00057)	*CSNK1G2*, *CDH10*
	VEGF signaling pathway (P00056)	*KDR*, *PIK3CD*
	Regulation of transcription by the bZIP transcription factor (P00055)	*POLR2E*, *MTERF2*
	Transcription regulation (P00023)	*POLR2E*, *MTERF2*
	FGF signaling pathway (P00021)	*PIK3CD*, *FGF22*
	PDGF signaling pathway (P00047)	*PIK3CD*, *PDGFRA*
	Circadian clock (P00015)	*CLOCK*, *CRY1*
LM	Integrin signaling pathway (P00034)	*ITGAV*, *LIMS2*
	PDGF signaling pathway (P00047)	*NCK1*, *RAB2A*
	CCKR signaling map (P06959)	*LYN*, *ITGAV*
HH	Inflammation involving chemokine and cytokine signaling pathway (P00031)	*SOCS5*, *RAC2*, *PIK3CD*, *MYH9*
	Angiogenesis (P00005)	*KDR*, *PIK3CD*, *PDGFRA*
	VEGF signaling pathway (P00056)	*KDR*, *RAC2*, *PIK3CD*
	Ras signaling pathway (P04393)	*RAC2*, *PIK3CD*, *RALB*
	FGF signaling pathway (P00021)	*RAC2*, *FGF12*, *PIK3CD*
	Notch signaling pathway (P00045)	*LNX1*, *HES1*, *MFNG*
	Integrin signaling pathway (P00034)	*RAC2*, *PIK3CD*
	PDGF signaling pathway (P00047)	*PIK3CD*, *PDGFRA*
CH	Interleukin signaling pathway (P00036)	*STAT6*, *STAT2*, *ELK3*, *IL23A*
	EGF receptor signaling pathway (P00018)	*STAT6*, *STAT2*, *ERBB3*
	DNA replication (P00017)	*PRIM1*, *H3F3C*, *Histone H3.3C*
	PDGF signaling pathway (P00047)	*STAT6*, *STAT2*, *RAB2A*
	JAK / STAT signaling pathway (P00038)	*STAT6*, *STAT2*
	Integrin signaling pathway (P00034)	*ITGA7*, *NTN4*
	Wnt signaling pathway (P00057)	*CSNK1G3*, *SMARCC2*
MO	Angiogenesis (P00005)	*KDR*, *PIK3CD*, *MAPK14*, *PDGFRA*
	p53 pathway feedback loop two (P04398)	*PIK3CD*, *MAPK14*, *MAPK13*, *CDKN1A*
	VEGF signaling pathway (P00056)	*KDR*, *PIK3CD*, *MAPK14*
	Ras signaling pathway (P04393)	*PIK3CD*, *MAPK14*, *MAPK13*
	FGF signaling pathway (P00021)	*PIK3CD*, *MAPK14*, *MAPK13*
	TGF-beta signaling pathway (P00052)	*MAPK14*, *MAPK13*, *TGFB2*
	EGF receptor signaling pathway (P00018)	*PIK3CD*, *MAPK14*, *MAPK13*
	Activation of B cells (P00010)	*PIK3CD*, *MAPK14*, *MAPK13*
	Gonadotropin-releasing hormone receptor pathway (P06664)	*MAPK14*, *MAPK13*, *TGFB2*
	Interleukin signaling pathway (P00036)	*FOXO3*, *CDKN1A*
	Integrin signaling pathway (P00034)	*PIK3CD*, *MAPK13*
BG	Wnt signaling pathway (P00057)	*SFRP5*, *BTRC*, *CSNK1G2*, *CDH1*
	Activation of B cells (P00010)	*LYN*, *CHUK*, *RAC2*, *NFKB2*
	Apoptosis signaling pathway (P00006)	*CHUK*, *MAP4K3*, *NFKB2*
	Inflammation involving chemokine and cytokine signaling pathway (P00031)	*CHUK*, *RAC2*, *NFKB2*
	Activation of T cells (P00053)	*CHUK*, *RAC2*, *NFKB2*
	FGF Signaling pathway (P00021)	*RAC2*, *FGF22*, *FGF8*
	Nicotinic acetylcholine receptor signaling pathway (P00044)	*CHRNA4*, *BCHE*, *ACTR1A*
	Gonadotropin-releasing hormone receptor pathway (P06664)	*RGS19*, *OPRL1*
RP	FGF signaling pathway (P00021)	*SPRY1*, *FGF2*, *PIK3CD*
	Opioid proenkephalin pathway (P05916)	*OPRK1*, *VAMP3*, *PDYN*
	Opioid proencephalin pathway (P05915)	*PENK*, *VAMP3*, *PDYN*
	Angiogenesis (P00005)	*FGF2*, *PIK3CD*
	Integrin signaling pathway (P00034)	*TLN1*, *PIK3CD*
	Wnt signaling pathway (P00057)	*CTNNA2*, *TLE4*
ZR	Inflammation involving chemokine and cytokine signaling pathway (P00031)	*CHUK*, *NFKB2*, *INPPL1*
	Activation of B cells (P00010)	*LYN*, *CHUK*, *NFKB2*
	Gonadotropin-releasing hormone receptor pathway (P06664)	*PTGER2*, *SNRPB*, *ADCY1*
ZB	Activation of B cells (P00010)	*LYN*, *SYK*, *CHUK*, *RAC2*, *PIK3CD*, *NFKB2*
	Inflammation involving chemokine and cytokine signaling pathway (P00031)	*CHUK*, *STAT1*, *RAC2*, *PIK3CD*, *NFKB2*
	PDGF signaling pathway (P00047)	*CHUK*, *STAT1*, *PIK3CD*, *STAT4*, *PDGFRL*
	Interleukin signaling pathwau (P00036)	*CHUK*, *STAT1*, *CSF2RB*, *STAT4*
	Activation of T cells (P00053)	*CHUK*, *RAC2*, *PIK3CD*, *NFKB2*
	EGF receptor signaling pathway (P00018)	*STAT1*, *RAC2*, *PIK3CD*, *STAT4*

## Supplementary Materials

The following are available online at https://www.mdpi.com/2076-2615/9/12/1024/s1, Supplementary Material S1. Characteristics of conserved breeds maintained in Poland; Supplementary Material S2. List of genes identified in breeds of cattle maintained in Poland (References [61,62] are cited in the Supplementary Materials).
