# Supplementary material for: A Comprehensive Analysis of Runs of Homozygosity of Eleven Cattle Breeds Representing Different Production Types
Source: Animals (Basel). 2019 Nov 25;9(12):1024. doi: 10.3390/ani9121024 (PMC6941163; doi:10.3390/ani9121024)
Supplement: Supplementary file 1 [file animals-09-01024-s001.pdf]

Supplementary Material S1. Characteristics of conserved breeds maintained in Poland.

Nowadays, breeding is dominated by usage of modern, high-production animal breeds, which despite their extraordinary production abilities are characterized by relatively poor health and/or deepening fertility problems [61]. Problems with longevity and health of highly selected animal breeds are associated, among others, with the genetic effect of the selection process, manifested in a decrease in genetic variability, especially in regions of the genome undergoing strong selection pressure [62]. Thus, it is necessary to maintain the genetic reserve, taking into account the most diverse and primitive animal breeds, characterized by a high level of genetic variation. In Poland, as a genetic reserve serve four cattle breeds: White-Backed (BG), Polish Red (RP), Polish Red-and-White (ZR) and Polish Black-and-White (ZB).

In general, Polish native cattle breeds are characterized by high resistance, health, longevity and very good fertility or features that were lost in intensively used breeds. In order to prevent the complete elimination of these breeds from animal production, programs for the protection of genetic resources were formed. Although the number of animals raised under the programs is currently a stable basis for further breeding, it should be noted that these breeds have been reproduced from a small number of animals, which may raise concerns about the structure and genetic variability of their present population. Polish native cattle breeds are kept as a dual-purpose milk-meat type.

All of the information presented below is derived from http://bydlo.bioroznorodnosc.izoo.krakow.pl/ and "Program for the Conservation of Genetic Resources of Farm Animals".

White-Backed cattle

White-Backed cattle origin from primitive cattle that inhabited north-east Europe and are characterized by a white belt, narrow at the withers and widening towards the croup, where it covers the entire width of the pelvis. The edge of the colored rim is irregularly jagged. White-Backed cattle survived both in Poland and Lithuania.

The height of an adult cow is about 130 cm, the adult bull about 140 cm

Average milk yield around 4000 kg per lactation, with a fat content of 3.8–4.0%, and protein content of 3.2–3.4%.

Number of dairy herds as for 2019-59. Number of dairy animals as for 2019-668.

Introduced to the "Program for the Conservation of Genetic Resources of Farm Animals" in 2004.

Polish Red

Polish red cattle are derived from small wild brachyceric (short-horned) cattle, living in eastern central Europe and Scandinavia. The beginnings of breeding of Polish Red cattle dates back to the second half of the 19th century, when herds of this breed were established in Polish lands, especially in Galicia. At the end of the 19th century systematic breeding work has been started, which lasts continuously until today.

Polish red cattle is one of the few indigenous European red cattle breeds. It is characterized by properties characteristic of indigenous populations, such as: high immunity, good health properties, longevity, very good fertility, light births, high calf vitality and ease of rearing, as well as high biological value of milk. It is also important that this cattle perfectly adapted to harsh environmental conditions and unrefined selection of fodder. Characteristics of construction of these cattle are strong legs and hooves, which means that cattle of this breed is well adapted to mountain living and production conditions. In addition, it is distinguished by important milk characteristics: high content of protein, fat and dry matter, high biological value and high suitability for cheese making purposes.

The height of an adult cow is about 135 cm, the adult bull about 140 cm.

Average milk yield around 4000–4500 kg per lactation, with a fat content of 4.5%, and protein content of 3.5%.

Number of dairy herds as for 2019-257. Number of dairy animals as for 2019-2513. Number of herds maintained for meat production as for 2019-54. Number of animals maintained for meat production as for 2019-773.

Introduced to the "Program for the Conservation of Genetic Resources of Farm Animals" in 1999.

Polish Red-and-White (ZR)

Red and white cattle have been known in Western Europe since around the 13th century. In the first half of the 17th century, this breed constituted the dominant part of the cattle population kept in the lowlands of the entire continent. The breeding of red and white cattle in the current Polish lands has been carried out for over 100 years. Cattle were brought to Poland from Westphalia, Rhineland and East Frisia, initially to the region of Lower Silesia and the Opole region, and later to the territory of Southern Poland.

Decades of being maintained in Polish conditions led to the creation of a breed resistant and well adapted to national conditions. Literature data also shows that red and white cattle were characterized by high resistance and easy adaptation to environmental conditions. Most likely, these traits, so extremely useful in difficult mountain and foothill conditions, determined the territorial range of the breed's occurrence. Currently, herds of this breed are mostly maintained along the southern border of Poland which is rich in mountainous areas.

The height of an adult cow is about 134–138 cm, the adult bull about 133–142 cm.

Average milk yield around 4500–5000 kg per lactation, with a fat content above 4.0%, and protein content of 3.3%.

Number of herds as for 2019-326. Number of animals as for 2019-3378.

Introduced to the "Program for the Conservation of Genetic Resources of Farm Animals" in 2008.

Polish Black-and-White (ZB)

Polish black and white cattle are characterized by features typical of indigenous populations, such as high resistance and health, longevity, as well as excellent adaptation to difficult environmental conditions and inaccessibility in the selection of feed. It is bred in lowland areas throughout Poland, except for the foothill regions of southwest, southwest and northeastern Poland.

The height of an adult cow is about 125–135 cm, the adult bull about 130–140 cm.

Average milk yield around 4000 kg per lactation, with a fat content of 4.03%, and protein content of 3.2%.

Number of herds as for 2019-108. Number of animals as for 2019–1556.

Introduced to the "Program for the Conservation of Genetic Resources of Farm Animals" in 2008.

Supplementary Material S2. List of genes identified in breeds of cattle maintained in Poland.

Breed	Genes
HO	ABCC5, ABCF3, ALG3, AP2M1, AQP9, ARL8B, ARPP19, B3GNT5, BHLHE40, BOLA, C16H1orf74, C20H5orf34, C9, CAMK1G, CAMK2N2, CCL28, CENPW, CHAC1, CLCN2, CRBN, CYP19A1, DAB2, DCUN1D1, DIEXF, DLL4, DNAAF4, DTWD1, ECE2, EDEM1, EGFLAM, EPHB3, FAM131A, FAU, FGF10, FGF7, FYB1, G0S2, GABPB1, GDNF, GHR, GNB5, HCN1, HDC, HMGCS1, HSD11B1, HTR3C, INO80, IRF6, ISL1, ITGA2, ITPR1, KCNH1, KLHL24, LAMB3, LAMP3, LEO1, LIFR, LOC503858, LOC524771, LOC788201, LRRN1, LYSMD2, MAGEF1, MAP6D1, MCCC1, MIR1224, MIR1248-1, MIR1248-2, MIR205, MIR2284Y-3, MIR628, MNS1, MOCS2, MRPS30, NIM1K, NNT, ONECUT1, OSMR, PAIP1, PARL, PARP8, PELO, PIGB, POLR2H, POLR2M, PSMD2, RAB27A, RFX7, RHOV, RICTOR, RSL24D1, RSPO3, SCG3, SELENOP, SERTAD4, SETMAR, SLC27A2, SPINT1, SUMF1, TCF12, TEX9, THPO, TMEM267, TMOD2, TMOD3, TRAF3IP3, TRH, TRNT1, TRPM7, UNC13C, USP50, USP8, VPS18, WDR70, YEATS2, ZNF131

RW	ABCC5, ABCF3, ACSF3, ACTR1A, ALG3, AMFR, AP2M1, APRT, ARL15, ARL2, ARL2BP, ASRGL1, ATG2A, ATL3, B3GAT3, B3GNT5, BAD, BATF2, BBS2, BEST1, BREH1, BSCL2, BTRC, C18H16orf78, C18H16orf87, C20H5orf34, C29H11orf84, C6, C7, C9, CAMK2N2, CAPN1, CARD6, CBLN1, CCDC88B, CCL22, CCL28, CDC42BPG, CDCA5, CDH15, CDK10, CENPW, CES1, CES5A, CHMP1A, CHRM1, CLCN2, COX8A, CPNE2, CPNE7, CSKMT, CTU2, CUEDC2, CYBA, DAB2, DAGLA, DBNDD1, DCUN1D1, DEF8, DGAT1, DNAJA2, DPCD, DPEP1, ECE2, EEF1G, EGFLAM, EHD1, ELOVL3, EML3, EPHB3, ESRRA, FADS2, FADS3, FAM131A, FAM192A, FANCA, FAU, FBXL15, FBXO4, FBXW4, FEN1, FERMT3, FGF10, FGF8, FKBP2, FST, FTH1, FYB1, GALNS, GANAB, GAS8, GBF1, GHR, GNAO1, GNG3, GPHA2, GPR137, GPT2, HCN1, HERPUD1, HIF1AN, HMGCS1, HNRNPUL2, HPS6, HRASLS5, HSPB3, HTR3C, IMPAD1, INTS5, IRX5, IRX6, ISL1, ITFG1, ITGA2, KCNIP2, KLHL24, LAMP3, LBHD1, LBX1, LIFR, LOC508916, LOC524771, LOC618367, LONP2, LPCAT2, LRRN4CL, LZTS2, MACROD1, MAGEF1, MAP4K2, MAP6D1, MARK2, MC1R, MCCC1, MEN1, MFSD13A, MGEA5, MIR1224, MIR138-2, MIR146B, MIR192, MIR194-2, MIR2284Y-3, MIR2285G-1, MIR2285N-3, MIR2327, MIR2406, MMP2, MOCS2, MRPL43, MRPL49, MRPS30, MT1A, MT1E, MT2A, MT3, MT4, MTA2, MVD, N4BP1, NAA40, NAALADL1, NDUFB8, NDUFS4, NFKB2, NIM1K, NNT, NOLC1, NPM3, NRXN2, NUDT21, NUDT22, NUP93, NXF1, OGFOD1, ORC6, OSMR, OTUB1, OXCT1, PAIP1, PARL, PARP8, PELO, PITX3, PLA2G16, PLCB3, PLCXD3, PLLP, POLL, POLR2G, POLR2H, PPP2R5B, PPRC1, PRDX5, PRKAA1, PSD, PSMD2, PTGER4, PYGM, RAB3IL1, RASGRP2, RCOR2, RICTOR, RNF166, ROM1, RPL13, RPL37, RPS6KA4, RSPO3, RSPRY1, RTN3, SAC3D1, SCGB1A1, SCGB1D, SCGB2A2, SEC31B, SELENOP, SEMA4G, SF1, SFXN3, SHCBP1, SIAH1, SLC12A3, SLC22A10, SLC22A31, SLC22A6, SLC22A8, SLC22A9, SLC3A2, SLC6A2, SLF2, SNAI3, SNX15, SNX18, SPATA2L, SPG7, SPIRE2, STIP1, STX5, SUFU, SYVN1, TAF6L, TCF25, THPO, TLX1, TM7SF2, TMEM179B, TMEM223, TMEM258, TMEM262, TMEM267, TRAPPC2L, TRH, TRMT112, TRPT1, TTC33, TTC9C, TUBB3, TUT1, TWNK, UBXN1, UQCC3, VEGFB, VPS35, VPS51, WDR74, YEATS2, ZFPL1, ZNF131, ZNF423, ZNHIT2
SM	ABCA7, ABCG2, ABHD17A, AFF4, ANGPTL7, ANKS1B, ARID3A, ASZ1, ATP10D, ATP5F1D, ATP8B3, BPIFC, BSG, BTBD11, BTNL9, BTNL9, C7H19orf24, C7H19orf25, CA6, CDC34, CDH10, CENPS, CEP135, CFD, CFTR, CHIC2, CIRBP, CLOCK, CNGA1, CNN2, CORT, CRY1, CSNK1G2, CTNNBIP1, CWH43, DANCR, DAZAP1, DCAF16, DCUN1D4, DFFA, DISP3, EFNA2, EIF3E, ELANE, EMC2, ENO1, ERRFI1, EXOC1, EXOSC10, FAM13A, FBXO2, FBXO44, FBXO6, FBXO7, FGF22, FIP1L1, FRYL, FSTL3, GAMT, GDF9, GPX4, GPX4, GPX4, GSX2, HERC3, HERC5, HERC6, HMHA1, HSPA4, IBSP, IFI47, KCNIP4, KDR, KIT, LAP3, LCORL, LEAP2, LNX1, LRRC66, LYPD8, MADCAM1, MASP2, MBD3, MBD3, MED28, MEPE, MGC137030, MIER2, MIR218-1, MIR34A, MIR4449, MIR6120, MISP, MTERF2, MUM1, NAP1L5, NCAPG, NDUFS7, NFXL1, NIPAL1, NLRP3, NMNAT1, NMU, OCIAD1, OCIAD2, PACRGL, PARK7, PDCL2, PDGFRA, PEX14, PGD, PIGY, PIK3CD, PKD2, PLPP2, POLR2E, POLRMT, PPM1K, PRSS57, PTBP1, PTBP1, PWP1, PYURF, R3HDM4, RACK1, RASL11B, REEP6, REXO1, RFX4, RNF126, RPS15, RSPO2, RTCB, SBNO2, SCAMP4, SCFD2, SGCB, SH3BP5L, SHROOM1, SLAIN2, SLC10A4, SLC2A5, SLIT2, SPP1, SPSB1, SRD5A3, SRM, SS18L2, SYN3, TARDBP, TARP, TBC1D5, TCF3, TECRL, TMEM165, TMEM201, TMEM263, TNFRSF9, TRIM41, TRIM52, TRIM7, TXK, UBE4B, UFM1, UQCR11, UQCRQ, USP46, VAMP3, WDR18, ZAR1, ZCCHC10, ZNF496, ZNF692
LM	AHSG, AMMECR1L, APH1B, ARHGEF4, ASNSD1, ASPH, ATP6V1H, BIN1, C2H2orf88, CA8, CALCRL, CCDC115, CHCHD7, CLVS1, COL3A1, CRYGS, CYFIP1, CYP7A1, DNAJB11, EIF4A2, ERCC3, FAM110B, FAM168B, FAM171B, FAM96A, FBXL22, FETUB, GPR17, GULP1, HERC1, HERC2, HRG, HS6ST1, IL20RB, IMP4, IMPAD1, INPP1, ITGAV, KNG1, KNG1, LACTB, LGSN, LHFPL3, LIMS2, LYN, LYPLA1, MASP1, MIR1248-1, MIR1248-2, MIR190A, MIR2285AA, MIR2285K-3, MIR2350, MIR2351, MIR2917, MRPL15, MSTN, NAB1, NCK1, NIPA2, NPBWR1, OPRK1, OPRK1, ORC5, ORMDL1, OSGEPL1, PCCB, PCMTD1, PENK, PLEKHB2, PMS1, POLR2D, POLR2K, PPIB, PROC, PTPN18, RAB2A, RAB8B, RB1CC1, RFC4, RGS20, RP1, RPS20, RPS27L, RTP1, SDCBP, SDR16C5, SDR16C6, SLC35G2, SLC40A1, SNX1, SNX22, SOX17, ST6GAL1, TBCCD1, TCEA1, TFPI, TGS1, TMEM68, TOX, TPM1, TPM1, TUBGCP5, UBXN2B, USP3, WDR33, WDR75, ZC3H15, ZSWIM2
HH	ACSS2, ADAMTS12, AHCY, ALG10, AMN1, ATP10D, C1QL2, C1QTNF3, C1QTNF6, C20H5orf22, C2H2orf76, CA6, CACNG2, CCDC50, CCDC93, CDH6, CENPS, CEP135, CHIC2, CLOCK, CNGA1,

	CORT, COX7B2, CPN2, CPNE1, CSF2RB, CTNNBIP1, CTPS1, CWH43, CYTH4, DANCR, DBI, DCUN1D4, DENND5B, DFFA, DNM1L, DYNLRB1, EDEM2, EIF3D, EIF6, EPB41L5, ERGIC3, EXOC1, EXOSC10, FGF12, FIP1L1, FOXRED2, FRYL, GABRA2, GABRA2, GABRA4, GABRB1, GABRG1, GABRG1, GDF5, GGT7, GOLPH3, GSS, GSX2, HES1, HOPX, HRASLS, HRASLS, IFT27, IGFBP7, INHBB, ITCH, KCTD17, KDR, KIAA1551, KIT, LNX1, LOC536190, LRRC66, MAP1LC3A, MASP2, MB21D2, MFNG, MGC148692, MIR1835, MIR34A, MIR4449, MIR499, MPST, MPST, MYH9, NCF4, NFS1, NFXL1, NIPAL1, NMNAT1, NMU, NOA1, NPR3, OCIAD1, OCIAD2, OPA1, OSTN, PAICS, PDCL2, PDGFRA, PEX14, PGD, PHF20, PIK3CD, PKP2, POLR2B, PPAT, PROCR, PTPN4, PVALB, RAC2, RALB, RASL11B, RBM12, RBM39, REST, ROMO1, SCAND1, SCFD2, SCMH1, SCTR, SGCB, SINHCAF, SLAIN2, SLC10A4, SLC2A5, SOCS5, SPAG4, SPINK2, SPSB1, SRD5A3, SRM, SRP72, STEAP3, SUB1, SYT10, TARDBP, TARS, TEX33, TMEM165, TMEM177, TMEM185B, TMEM201, TMEM37, TRPC4AP, TST, TXK, TXN2, UBE4B, UFM1, UQCC1, USP18, USP46, YARS2, ZAR1
CH	ABCG2, AGAP2, ALDH7A1, AMDHD1, ANKRD52, APOF, ApoN, ARHGEF25, ASPH, ATP23, ATP5F1B, ATP6V1H, AVIL, B4GALNT1, BAZ2A, BLOC1S1, CA8, CD63, CDK2, CDK4, CEBPD, CEP120, CHCHD7, CLVS1, CNPY2, COQ10A, CS, CSNK1G3, CTDSP2, CYP27B1, CYP7A1, DCAF16, DCTN2, DDIT3, DGKA, DNAJC14, DTX3, EEF1AKMT3, EFCAB1, ELK3, ERBB3, ESYT1, FAM110B, FAM13A, GLI1, GLS2, GPR182, GRAMD2B, H3F3C, HAL, HAS2, HERC3, HERC5, HERC6, HSD17B6, IBSP, IKZF4, IL23A, IMPAD1, INHBC, INHBE, ITGA7, ITGA7, KIF5A, LAP3, LCORL, LMNB1, LOC507581, LOC507581, LOC768323, LOC787518, LOC788615, LRIG3, LTA4H, LYN, LYPLA1, MARCH3, MARS, MCM4, MED28, MEPE, METTL1, MIP, MIR1251, MIR135A-2, MIR2430, MIR2431, MIR2432, MIR2433, MIR2458, MIR26A-2, MIR677, MMP19, MRPL15, MYL6, MYL6B, MYO1A, NAB2, NABP2, NACA, NAP1L5, NCAPG, NDUFA4L2, NEDD1, NEMP1, NPBWR1, NTN4, OPRK1, OPRK1, ORMDL2, OS9, OTX1, PA2G4, PAN2, PCMTD1, PENK, PHAX, PIGY, PIP4K2C, PKD2, PMEL, POLR2K, PPDPFL, PPM1K, PRDM6, PRIM1, PRKDC, PTGES3, PYM1, PYURF, R3HDM2, RAB2A, RAB5B, RB1CC1, RBMS2, RDH16, RDH5, RGS20, RNF41, RP1, RPS20, RPS26, SARNP, SDCBP, SDR16C5, SDR16C6, SDR9C7, SHMT2, SLC16A7, SLC26A10, SLC39A5, SMARCC2, SNAI2, SNRPF, SOX17, SPP1, SPRYD4, STAC3, STAT2, STAT6, SUOX, TAC3, TCEA1, TEX43, TGS1, TIMELESS, TMEM17, TMEM68, TOX, TSFM, TSPAN31, UBE2V2, UBXN2B, WDPCP, ZC3H10, ZNF608
MO	ABCG2, ABHD1, AFG1L, AGBL5, ARMC2, ASCC3, ATG5, ATG5, ATP2C1, ATRAID, BEND3, BRPF3, BRWD1, BRWD1, C9H6orf203, CA6, CAD, CD164, CDC40, CDKN1A, CENPA, CENPS, CEP135, CEP57L1, CGREF1, CHIC2, CLOCK, CLPS, CORT, CPNE5, CTNNBIP1, DCAF16, DDO, DEF6, DFFA, DNAJC11, DNAJC5G, DPYSL5, EIF2B4, EMILIN1, ENO1, ERRFI1, EXOC1, EXOSC10, FAM13A, FANCE, FIG4, FIP1L1, FKBP5, FNDC4, FOXO3, GOLIM4, GPR6, GRIK2, GSX2, HERC3, HERC5, HERC6, HMGN1, IBSP, IFI47, IGSF5, IGSF5, IGSF5, KCTD20, KDR, KHK, KIT, KRTCAP3, LAP3, LCA5L, LCORL, LHFPL5, LNX1, LOC534913, LOC786597, LRRC77, LYPD8, MAPK13, MAPK14, MAPRE3, MASP2, MED28, MEPE, METTL24, MGC137030, MICAL1, MIR2285W, MIR2288, MIR34A, MIR551B, MPV17, MRPL3, NAP1L5, NCAPG, NLRP3, NMNAT1, NMU, NR2E1, NRBP1, NUDT16, OSTM1, PARK7, PCP4, PDCD10, PDCL2, PDGFRA, PDSS2, PEX14, PGD, PIGY, PIK3CD, PKD2, PNPLA1, PPARD, PPIL6, PPM1G, PPM1K, PRDM1, PREB, PSMG1, PYURF, QRSL1, RPL10A, RPS4Y1, RRP15, RTN4IP1, SCFD2, SCML4, SEC63, SERPINI1, SERPINI2, SESN1, SH3BGR, SH3BP5L, SIM1, SLC26A8, SLC2A5, SLC30A3, SLC35F6, SLC45A1, SLC5A6, SMPD2, SNX17, SNX3, SOBP, SPP1, SPSB1, SRD5A3, SRM, SRSF3, STK38, TARDBP, TCF23, TCP11, TGFB2, TMEM165, TMEM201, TMEM214, TNFRSF9, TRIM54, TULP1, UBE4B, UCN, UFM1, VAMP3, WASF1, WRB, ZBTB24, ZNF496, ZNF512, ZNF513, ZNF692
BG	ABCA7, ABHD16B, ABHD17A, ACTR1A, AFF4, ALG10, ARFGAP1, ARFRP1, ARID3A, ARID4A, ARL3, AS3MT, ATL2, ATP5F1D, ATP5MD, ATP8B3, AVPI1, BCHE, BMP2, BSG, BTRC, C13H20orf196, C1QTNF6, C7H19orf24, C7H19orf25, CALHM2, CALHM3, CDC34, CDH1, CDKL4, CDS2, CFD, CHCHD7, CHGB, CHRNA4, CHTF8, CHUK, CIRBP, CNN2, CNNM1, CNNM2, COG8, COX15, CPN1, CRLS1, CRTAC1, CRTAC1, CSNK1G2, CUEDC2, CUTC, CWF19L1, CYB5B, CYP17A1, CYP7A1, CYTH4, DAAM1, DAZAP1, DGAT1, DHX57, DNAJC5, DNAJC5, DNMBP, DPCD, EEF1A2, EFNA2, ELANE, ELOVL3, ERLIN1, EXOSC1, FAM110B, FBXL15, FBXW4, FERMT1, FGF22, FGF8, FNDC11, FSTL3, GALM, GAMT, GBF1, GDF9, GEMIN6, GID8, GINS1, GMEB2, GOLGA7B, GOT1, GPX4, GPX4, GPX4, HAS3, HIF1AN, HMHA1, HNRNPLL, HOGA1, HPS1, HPS6,

	HSPA4, IMPAD1, INA, KCNIP2, KCTD17, LBX1, LEAP2, LOC511498, LOXL4, LOXL4, LYN, LYPD8, LZTS2, MADCAM1, MAP4K3, MARVELD1, MBD3, MBD3, MCM8, MFNG, MFSD13A, MGC137030, MGEA5, MIER2, MIR124B, MIR1287, MIR1307, MIR1388, MIR140, MIR146B, MIR1835, MIR2284Z-2, MIR2392, MIR2393, MIR6120, MISP, MMS19, MORN2, MORN4, MPST, MPST, MRPL43, MUM1, MYT1, NDUFB8, NDUFS7, NEURL1, NFKB2, NIP7, NKX2-3, NOB1, NOLC1, NPBWR2, NPM3, NQO1, NT5C2, OPRL1, OPRL1, PCGF6, PCMTD2, PCNA, PDCD11, PDYN, PENK, PGAM1, PI4K2A, PITX3, PLPP2, POLL, POLR2E, POLRMT, PPDPF, PPRC1, PROKR2, PRPF6, PRSS57, PSD, PSMD7, PTBP1, PTBP1, PTK6, PYROXD2, R3HDM4, RAC2, REEP6, REXO1, RGS19, RHOBTB1, RNF126, RPS15, RPS20, RRP12, RTEL1, RTEL1, SAMD10, SBNO2, SCAMP4, SCD, SDCBP, SDR16C5, SDR16C6, SEC31B, SEMA4G, SFRP5, SFXN2, SFXN3, SH3BP5L, SHROOM1, SI, SIRPA, SIRPB1, SLC17A9, SLC23A2, SLC25A28, SLF2, SNTB2, SORCS3, SOX18, SRMS, SRSF7, STMN3, SUFU, SYT10, TAF5, TCEA2, TCF3, TERF2, TERF2, TEX33, TGS1, TIMM9, TLX1, TMED6, TMEM230, TMEM68, TNFRSF6B, TPD52L2, TRIM8, TRMT6, TST, TWNK, UBTD1, UBXN2B, UQCR11, UQCRQ, USP18, UTP4, VPS4A, WDR18, WWP2, YTHDF1, ZBTB46, ZCCHC10, ZDHHC16, ZFYVE27, ZGPAT, ZNF692
RP	ABHD16B, ADRM1, AHSA2, ANGPTL7, ANP32B, ARFRP1, ARHGEF39, ATP6V1H, BBS12, CA6, CCDC107, CENPS, CETN4, CHCHD7, CHRNA4, CLDN8, CORO2A, CORT, CREB3, CTNNA2, CTNNBIP1, CYP7A1, DFFA, DGAT1, DISP3, DNAJB5, DNAJC5, EEF1A2, ENO1, ERRFI1, EXOSC10, EXOSC3, FAM110B, FAM166B, FAM205C, FAM214B, FANCG, FBXO10, FBXO2, FBXO44, FBXO6, FGF2, FNDC11, GATA5, GID8, GINS1, GMEB2, GRHPR, HEMGN, HINT2, IGFBPL1, IL2, IL21, IMPAD1, KRTAP13-1, LYN, LYPLA1, MASP2, MRGBP, MRPL15, MTG2, MYT1, NANS, NMNAT1, NPBWR2, NTSR1, NUDT6, OGFR, OPRK1, OPRL1, OSBPL2, PARK7, PCMTD2, PDYN, PENK, PEX14, PGD, PIK3CD, POLR1E, POLR2K, PPDPF, PRPF6, PSMA7, PTK6, REG3G, RGP1, RGS19, RGS20, RP1, RPS20, RPS21, RUSC2, SAMD10, SDCBP, SDR16C5, SDR16C6, SHB, SIRPA, SIRPB1, SIT1, SLC17A9, SLC45A1, SLCO4A1, SOX17, SOX18, SPAG8, SPRY1, SPSB1, SRM, SRMS, SS18L1, STK35, STMN3, STOML2, TARDBP, TBC1D2, TCEA1, TCEA2, TDRD7, TESK1, TGS1, TLE4, TLN1, TMEM201, TMEM68, TMEM8B, TMOD1, TNFRSF6B, TNFRSF9, TOMM5, TPD52L2, TPM2, TRMO, TRMT10B, TRPC3, UBE4B, UBXN2B, UNC13B, VAMP3, VCP, XPA, XPO1, YTHDF1, ZBTB46, ZBTB5, ZCCHC7, ZGPAT
ZR	ABHD16B, ACSF3, ACTR10, ACTR1A, ADARB2, ADCY1, ADRM1, ALG10, ANAPC15, APRT, ARFGAP1, ARFRP1, ARID4A, BTRC, C18H16orf87, CA8, CCDC175, CCM2, CDH15, CDK10, CDS2, CHCHD7, CHMP1A, CHRNA4, CHUK, CLPB, CNNM1, COX15, CPN1, CPNE7, CPXM1, CTU2, CUEDC2, CUTC, CWF19L1, CYBA, CYP7A1, DAAM1, DBNDD1, DDX56, DEF8, DGAT1, DIP2C, DNAJA2, DNAJC5, DNAJC5, DNMBP, DPCD, DPEP1, EEF1A2, ELOVL3, ERLIN1, FAM110B, FANCA, FBXL15, FBXO11, FBXW4, FGF8, FNDC11, FOLR1, FOLR2, FOLR3, FOLR3, FOLR3, FOLR3, GALNS, GAS8, GATA5, GBF1, GID8, GINS1, GMEB2, GOT1, GPT2, GTF2A1L, GTPBP4, H2AFV, HAO1, HIF1AN, HPS6, IDH3B, IDH3B, IDH3B, IDI1, IGFBP1, IGFBP3, IMPAD1, INPPL1, IRX5, IRX6, JKAMP, KCNIP2, L3HYPDH, LAMTOR1, LARP4B, LBX1, LHCGR, LOC511498, LPCAT2, LRRC51, LRRC51, LRRC51, LYN, LZTS2, MC1R, MFSD13A, MGEA5, MIR1-1, MIR124B, MIR133A-1, MIR133C, MIR1388, MIR146B, MIR2285M-2, MIR2306, MIR2317, MIR2327, MIR2420, MIR4657, MMP2, MRGBP, MRPL43, MSH6, MTFMT, MTG2, MVD, MYT1, NDUFB8, NFKB2, NKX2-3, NOLC1, NOP56, NPBWR2, NPM3, NTSR1, NUDCD3, NUMA1, OGDH, OGFR, OPRL1, OPRL1, ORC6, OSBPL2, PARL, PCMTD2, PCNA, PDYN, PENK, PHOX2A, PITX3, POLL, PPDPF, PPIA, PPP1R21, PPRC1, PRND, PRNP, PRNP, PRNP, PROKR2, PRPF6, PSD, PSMA3, PSMA7, PTGER2, PTK6, PURB, RAB2A, RAMP3, RASL12, RASSF2, RGS19, RNF121, RNF166, RPL13, RPS20, RPS21, RTEL1, RTEL1, SAMD10, SCD, SDCBP, SDR16C5, SDR16C6, SEC31B, SEMA4G, SFXN3, SHCBP1, SIRPA, SIRPB1, SLC17A9, SLC22A31, SLC23A2, SLC25A28, SLC51B, SLCO4A1, SLF2, SNAI3, SNRPB, SOX18, SPATA2L, SPG21, SPG7, SPIRE2, SPIRE2, SRMS, SS18L1, STK35, STMN3, STON1, SUFU, SYT10, TBRG4, TCEA2, TCF25, TGM3, TGS1, TIMM9, TLX1, TMC2, TMED4, TMEM230, TMEM68, TNFRSF6B, TOMT, TOX, TPD52L2, TRAPPC2L, TRPC2, TUBB3, TWNK, UBXN2B, USP18, VPS35, WDR37, YTHDF1, ZBTB46, ZGPAT, ZMIZ2, ZMYND11
ZB	ABCA7, ABCC5, ABCF3, ABHD17A, ACSF3, ACTR1A, AFF4, ALG10, ALG3, AP2M1, ARHGEF4, ARID3A, ASAH1, ATP5F1D, ATP6V1H, ATP8B3, AUH, AUH, B3GNT5, BMP2, BTRC, C1QTNF6, C7H19orf24, C7H19orf25, CAMK2N2, CCDC115, CDH15, CDK10, CENPS, CFD, CHCHD7, CHMP1A, CHUK, CIRBP, CLCN2, CNN2, CORT, COX15, CPN1, CPNE7, CRLS1, CSF2RB,

	CSNK1G2, CTNNBIP1, CUEDC2, CUTC, CWF19L1, CYFIP1, CYP7A1, CYTH4, DAZAP1, DBNDD1, DEF8, DFFA, DGAT1, DIRAS2, DNM1L, DNMBP, DPCD, DPEP1, ECE2, ECE2, ECE2, ECE2, ECE2, EFNA2, ELANE, ELOVL3, EPHB3, ERLIN1, FAM110B, FAM131A, FAM168B, FANCA, FBXL15, FBXW4, FERMT1, FGF8, FGL1, FRG1, FSTL3, GAMT, GAS8, GBF1, GCC2, GDF9, GLS, GPX4, GPX4, GPX4, GYPC, HERC2, HIF1AN, HMHA1, HPS6, HSPA4, HTR3C, IMP4, IMPAD1, KCNIP2, KCTD17, KLHL24, LBX1, LEAP2, LIMS1, LOC511498, LOC524771, LYN, LYPLA1, LZTS2, MAGEF1, MAP6D1, MBD3, MBD3, MC1R, MCM8, MFNG, MFSD13A, MGEA5, MIR1224, MIR146B, MIR1835, MIR2284AC, MIR2284Y-3, MIR2398, MIR6120, MISP, MNS1, MPST, MPST, MRPL15, MRPL43, MTMR7, MTUS1, MUM1, NCF4, NDUFB8, NDUFS7, NFIL3, NFKB2, NIPA2, NKX2-3, NMNAT1, NOLC1, NPBWR1, NPM3, OPRK1, OPRK1, ORC6, PARL, PDGFRL, PENK, PEX14, PGD, PIK3CD, PITX3, PKP2, PLEKHB2, POLL, POLR2E, POLR2H, POLR2K, POLR2M, PPRC1, PRSS57, PSD, PSMD2, PTBP1, PTBP1, PTPN18, R3HDM4, RAC2, RANBP2, RB1CC1, REEP6, REXO1, RFX7, RGS20, ROR2, RP1, RPL13, RPS15, RPS20, SBNO2, SCAMP4, SCD, SDCBP, SDR16C5, SDR16C6, SEC31B, SEMA4G, SEPT10, SFXN3, SHCBP1, SHROOM1, SLC22A31, SLC25A28, SLC5A7, SLF2, SOX17, SPATA2L, SPG7, SPIRE2, SPIRE2, SPSB1, STAT1, STAT4, SUFU, SULT1C3, SULT1C4, SYK, SYT10, TCEA1, TCF12, TCF25, TCF3, TEX33, TEX9, TGS1, THPO, TLX1, TMEM201, TMEM68, TRMT6, TST, TUBB3, TUBGCP5, TWNK, UBE4B, UBXN2B, UQCR11, UQCRQ, USP18, VPS35, WDR18, YARS2, YEATS2, ZCCHC10
